# Liposomal Vitamin C as a Modulator of the Efficacy of Ceralasertib Therapy in Ovarian Cancer

**DOI:** 10.3390/ijms27062630

**Published:** 2026-03-13

**Authors:** Patrycja Gralewska-Zając, Aleksandra Przybylska, Marek Langner, Magdalena Przybyło, Agnieszka Marczak, Aneta Rogalska

**Affiliations:** 1 Department of Medical Biophysics, Institute of Biophysics, Faculty of Biology and Environmental Protection, University of Lodz, 141/143 Pomorska Street, 90-236 Lodz, Poland; patrycja.gralewska.zajac@biol.uni.lodz.pl (P.G.-Z.); aleksandra.przybylska@edu.uni.lodz.pl (A.P.); agnieszka.marczak@biol.uni.lodz.pl (A.M.); 2 Department of Biomedical Engineering, Wroclaw University of Science and Technology, 13 Grunwaldzki Square, 50-377 Wrocław, Poland; marek.langner@lipid-systems.pl (M.L.); magdalena.przybylo@lipid-systems.pl (M.P.); 3 Lipid Systems Ltd., 48C Krzemieniecka Street, 54-613 Wrocław, Poland

**Keywords:** liposome, vitamin C, ceralasertib, ATR pathway, ferroptosis, ovarian cancer

## Abstract

Clinical evidence suggests that vitamin C (VitC) may enhance the efficacy of cancer chemotherapy. However, its high oxidating and reducing activity results in low stability in physiological fluids, which may compromise its supportive role in cancer therapies. VitC stability improves when located in a region where water activity is reduced and exposure to a limited amount of ferrous ions. This can be achieved when VitC is encapsulated in liposomes. Here, we present a novel combinatorial effect of a liposomal formulation of vitamin C (LVC, liposomal VitC) and an ataxia-telangiectasia and Rad3-related (ATR) kinase inhibitor (ATRi, ceralasertib) on cancer cells. The cytotoxic effects of vitamin C, LVC and ATRi were evaluated using spectrophotometric and spectrofluorimetric assays, flow cytometry and Western blot. Lipid peroxidation was assessed via fluorescence microscopy and quantified by spectrofluorimetric assays. DNA damage was examined by Western blot. The combination has higher efficacy than ceralasertib alone in genetically diverse ovarian cancer cell lines. LVC offers protective effects when used as an adjuvant during anticancer therapy. We found that the inhibition of the ATR pathway in the presence of LVC results in increased intracellular calcium levels, elevated lipid peroxidation, and higher Fe^2+^ concentrations. The upregulation of ROS, together with the increased expression of long-chain-fatty-acid—CoA ligase 4 (ACSL4) following co-treatment with ATRi and LVC, indicates the activation of ferroptotic pathways. The formation of DNA double-strand breaks suggests replication fork collapse. Our findings demonstrates that this synthetic targeted therapy, combining a novel liposomal formulation of VitC with an ATR inhibitor, not only enhances DNA damage and the cytotoxic efficacy of ceralasertib but also effectively drives ovarian cancer cells toward cell death.

## 1. Introduction

Ovarian cancer (OC) is one of the most aggressive gynecological malignancies, characterized by a persistently low 5-year survival rate of approximately 30%, a figure that has shown little improvement over the past three decades [1]. The vast majority of malignant ovarian tumors, estimated at about 90%, originate from epithelial cells. Epithelial ovarian cancer (EOC) encompasses several histopathological subtypes, including serous (42%), mucinous (12%), endometrioid (15%), undifferentiated (17%), and clear-cell carcinoma (6%) [2]. Prognosis in OC is determined by several factors, such as clinical stage, histological grade, residual tumor burden after surgery, and, more recently, the status of homologous recombination repair. Most patients are diagnosed at an advanced stage, when the disease has already disseminated to distant sites, which substantially limits therapeutic options. Current standard management consists of cytoreductive surgery followed by systemic chemotherapy [3]. Despite initial responsiveness to surgery and chemotherapies, most patients experience relapse and metastatic progression, which markedly worsens clinical outcomes and remains the principal cause of mortality in OC [4,5].

One area of growing interest is the role of redox regulation and iron metabolism in cancer biology. Vitamin C, a potent redox-active molecule, exerts dual functions depending on its concentration. At physiological levels, it reduces Fe^3+^ to Fe^2+^, supporting enzymatic processes such as DNA synthesis and epigenetic regulation. However, at high concentrations, vitamin C can fuel the Fenton reaction, leading to the overproduction of ROS and oxidative stress [6,7]. This mechanism is closely linked to ferroptosis, a recently identified, iron-dependent, regulated form of cell death characterized by lipid peroxidation and the production of excessive reactive oxygen species (ROS), distinguishing it from apoptosis and necrosis [8]. The execution of ferroptosis is primarily linked to glutathione (GSH) depletion and the inhibition of glutathione peroxidase 4 (GPx4), a key antioxidant enzyme. Moreover, as ferroptotic cell death is driven by the accumulation of iron-dependent lipid peroxides, and ACSL4, an enzyme that converts fatty acids into fatty acyl-CoA esters, has been identified as a critical regulator of this process [9].

Disruption of these defense systems results in unchecked lipid peroxidation and cell death [10]. By modulating both redox balance and iron availability, vitamin C has emerged as a potential regulator of ferroptotic pathways in cancer cells. Vitamin C has demonstrated promising synergistic effects with standard chemotherapeutics, including carboplatin and paclitaxel, in preclinical and clinical studies of ovarian cancer [11,12,13]. Its activity is mediated by hydrogen peroxide generation in the tumor microenvironment, leading to DNA damage, metabolic disruption, and enhanced chemosensitivity, while simultaneously alleviating chemotherapy-related toxicities such as neurotoxicity and organ damage. Although survival benefits remain inconclusive, vitamin C has been associated with prolonged progression-free intervals and improved patient quality of life, underscoring its value as a potential adjuvant therapy [12].

However, the therapeutic application of vitamin C is limited by its inherent instability. Environmental and physiological factors such as light, high temperature, enzymatic oxidation, atmospheric oxygen, metal ions, and alkaline pH accelerate its degradation, leading to reduced bioavailability and therapeutic effectiveness [14]. To overcome these limitations, liposomal vitamin C (LVC) formulations have been developed. In our liposomal formulation, vitamin C is preferentially located within the lipid bilayer interface, where water activity is reduced; hence, the extent of hydrolysis is also reduced [15]. By encapsulating ascorbate within phospholipid bilayers, LVC improves stability, protects against premature degradation, and enhances systemic absorption, often achieving higher and more sustained plasma concentrations than conventional forms, potentially broadening the therapeutic window of vitamin C in oncology [16].

In parallel, targeting the DNA damage response (DDR) has emerged as another promising approach. Ataxia telangiectasia and Rad3-related kinase (ATR) is a master regulator of the DNA damage response (DDR), orchestrating DNA replication, repair, and cell cycle checkpoints. Cancer cells, particularly those harboring *TP53* mutations and lacking a functional G1 checkpoint, are highly dependent on the ATR-mediated control of S and G2/M transitions [17,18]. ATR inhibitors (ATRi) disrupt checkpoint signaling and DNA repair, driving premature mitotic entry and mitotic catastrophe, and ultimately inducing cell death independently of p53 status [17,19]. This synthetic vulnerability makes ATR inhibition, alone or in combination with DNA-damaging agents, a compelling therapeutic strategy in ovarian cancer.

Taken together, these insights highlight the therapeutic potential of combining redox-modulating agents such as LVC with DDR-targeting strategies like ATR inhibition. Such approaches may not only enhance the cytotoxic effects on ovarian cancer cells but also improve treatment tolerability, offering a promising direction for future therapies.

## 2. Results

### 2.1. Synergistic Effects of ATRi and Liposomal Vitamin C in OC Cell Lines

**Figure 1:**
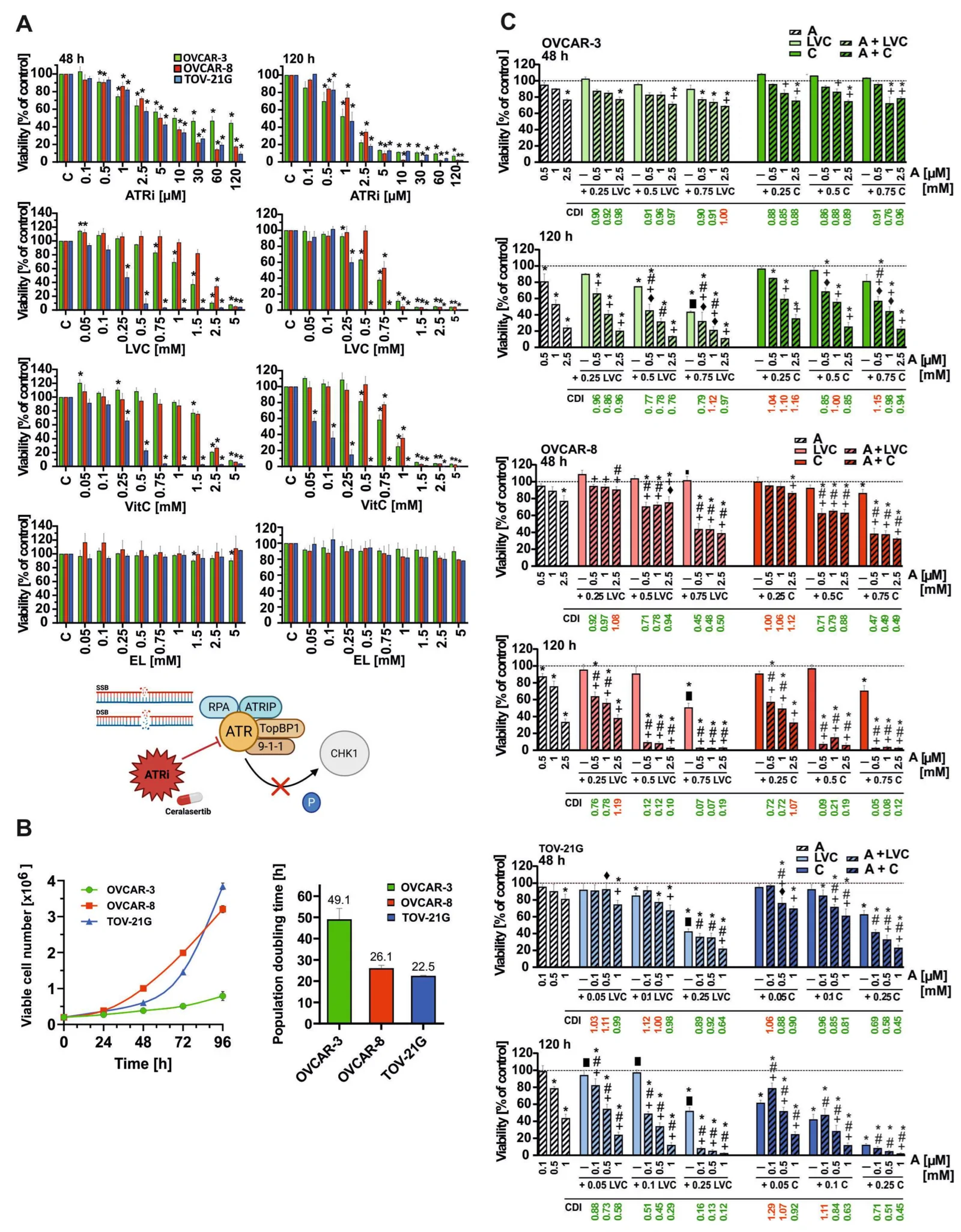
ATRi in combination with LVC decreased the viability of OC cells more effectively than ATRi alone. (**A**) Cell viability after treatment with ATRi, LVC, VitC or EL in OVCAR-3, OVCAR-8 and TOV-21G cell lines, at increasing concentrations for 48 h and 5 days, was assessed by the MTT assay (* *p* < 0.05). (**B**) Cell-doubling time was calculated from the cell growth curve during the exponential growth phase using the formula Td = t/log2 (Nt/N0). (**C**) The effect of ATRi combinations with LVC or VitC at different ratios, after 48 h and 5 days of treatment, was evaluated by MTT assay. * Statistically significant differences between cells incubated with the compound compared with the control cells (*p* < 0.05). # Statistically significant changes between cells incubated with ATRi and combination treatment (A + LVC; A + VitC) (*p* < 0.05). + Statistically significant differences between the cells incubated with LVC or VitC and combination treatment (A + LVC; A + VitC) (*p* < 0.05)). ❙ Statistically significant differences between cells incubated with the LVC compared to VitC (*p* < 0.05). ♦ Statistically significant changes between combination treatments (ATRi + LVC and ATRi + VitC) (*p* < 0.05).

Cytotoxicity assay after 48 and 120 h exposure revealed differential sensitivities of OVCAR-3, OVCAR-8, and TOV-21G cells to ATRi, liposomal vitamin C (LVC), and free vitamin C (VitC) (Figure 1A). The TOV-21G cell line displayed the highest sensitivity to the tested compounds. In OVCAR-8 cells, a reduction in viability to approximately 60% was observed at only 5 μM ATRi after 48 h treatment, whereas in TOV-21G, this effect occurred at 2.5 μM. At this concentration, no significant cytotoxicity was detected in OVCAR-8. For OVCAR-3 cells, a viability level of ~60% was also observed at 2.5 μM ATRi; however, further dose escalation led to a marked decrease in proliferation, to below 50% at 5 μM. A similar pattern was observed for both LVC and VitC treatments. In OVCAR-8 cells, a viability reduction to about 40% required 2.5 mM after 48 h treatment, whereas in TOV-21G the same effect was achieved at 0.25 mM, with 2 mM reducing viability to nearly zero (<3%). For OVCAR-3, a 60% reduction was noted at 1 mM, indicating intermediate sensitivity compared with the other cell lines. Further, dose increases caused additional viability declines, although these were less pronounced than in TOV-21G. At 48 h, IC_50_ values confirmed the lower sensitivity of OVCAR-3 to ATRi and comparable responses to LVC/VitC, as shown in Table 1. After 120 h, IC_50_ values decreased across all cell lines, most notably in OVCAR-3, indicating enhanced time-dependent sensitivity. Empty liposomes did not affect cell viability.

To assess potential synergistic interactions, cells were treated with ATRi, LVC, and VitC, alone or in combination, within concentration ranges tailored to each cell line (Figure 1C). Based on the CDI analysis, the lowest concentrations of the compounds to exhibit synergistic effects in combination treatment after 48 h of incubation (CDI < 1) were selected for further experiments (OVCAR-3: ATRi 2.5 µM, LVC 0.5 mM, VitC 0.5 mM; OVCAR-8: ATRi 2.5 µM, LVC 0.75 mM, VitC 0.75 mM; TOV-21G: ATRi 1 µM, LVC 0.25 mM, VitC 0.25 mM). At these concentrations, combination therapy significantly reduced viability compared to single agents. For instance, after 48 h, ATRi + VitC decreased viability to 32.7% in OVCAR-8 (CDI = 0.49) and 23.3% in TOV-21G (CDI = 0.45). OVCAR-3 remained less responsive, with around 70–79% viability after 48 h, though prolonged incubation (120 h) markedly enhanced cytotoxicity (20.6–23.8% viability). For OVCAR-8 and TOV-21G, five-day exposure reduced viability to below 4%. Moreover after 48 h, ATRi + LVC decreased viability to 39.1% in OVCAR-8 (CDI = 0.5), 21.13% in TOV-21G (CDI = 0.64) and 71.63 in OVCAR-3 (CDI = 0.97).

The cell-doubling time (DT) was determined to assess the proliferation rate of the analyzed cancer cell lines. Based on cell count analysis, the fastest population growth was observed for the TOV-21G (DT = 22.5 h) and OVCAR-8 (DT = 24 h) cell lines, compared with OVCAR-3 (49 h), consistent with differences in treatment sensitivity (Figure 1B).

### 2.2. ATR Inhibition Combined with LVC Modulates ROS and Glutathione Balance in Ovarian Cancer Cells

**Figure 2:**
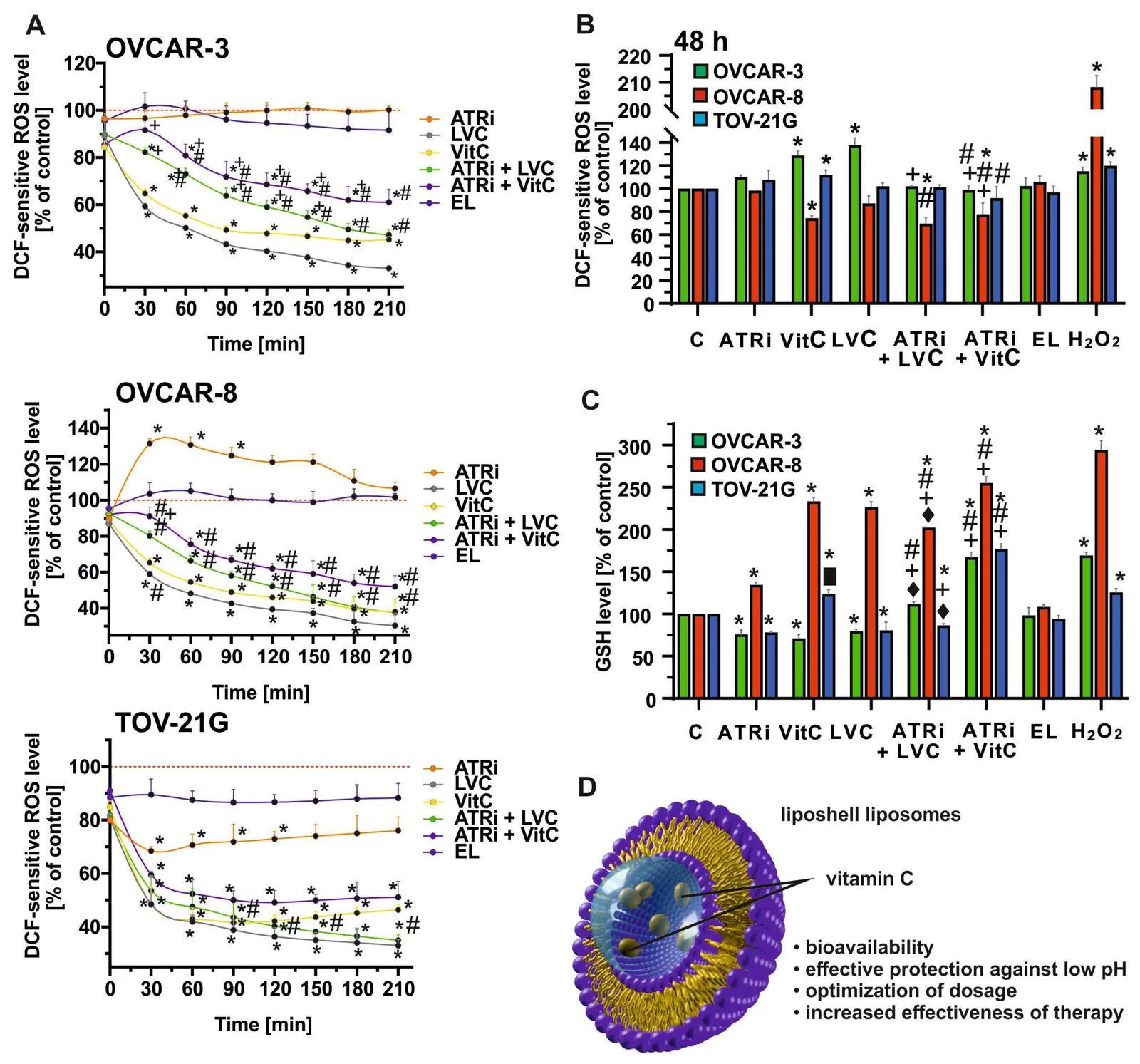
Analysis of intracellular ROS and glutathione levels in ovarian cancer cells after treatment. (**A**) Time-dependent changes in DCF-sensitive ROS production in OVCAR-3, OVCAR-8, and TOV-21G cell lines, measured over 210 min. (**B**) ROS levels measured after 48 h of treatment. (**C**) Intracellular glutathione (GSH) levels after 48 h of treatment. (**D**) LIPOSHELL^®^ liposomes spherical carriers with a diameter of 170 nanometers. They are composed of a double-lipid membrane filled with an aqueous phase. * Statistically significant differences between cells incubated with the compound compared with the control cells (*p* < 0.05). # Statistically significant changes between cells incubated with ATRi and combination treatment (A + LVC; A + VitC) (*p* < 0.05). + Statistically significant differences between the cells incubated with LVC or VitC and combination treatment (A + LVC; A + VitC) (*p* < 0.05). ❙ Statistically significant differences between cells incubated with the LVC compared to VitC (*p* < 0.05). ♦ Statistically significant changes between combination treatments (ATRi + LVC and ATRi + VitC) (*p* < 0.05).

To evaluate whether ATR inhibition in combination with LVC alters the redox balance of ovarian cancer cells, we first monitored the kinetics of dichlorofluoresein (DCF)-sensitive ROS levels by using 2′,7′-dichlorodihydrofluorescein diacetate (DCFH2-DA) probe (Figure 2A). In all cell lines, treatment with VitC or LVC alone caused an early but transient decrease in ROS, which then further declined over time. In contrast, combined treatments (ATRi + LVC, ATRi + VitC) led to a pronounced and sustained decrease in ROS levels compared with control and single agents; their levels were higher than LVC or VitC alone. ATR inhibition alone showed minimal effects, while exposure to EL did not affect ROS generation. Positive control experiments with H_2_O_2_ are shown in Supplementary Figure S1. To further validate these findings, ROS levels were quantified after 48 h of treatment (Figure 2B). Consistent with the short-term assays, VitC and LVC alone significantly increased ROS in OVCAR-3 and TOV-21G, while only a modest effect was observed in OVCAR-8. Importantly, the combination of ATR inhibition with either LVC or VitC markedly decreased ROS in all cell lines compared with single treatments, suggesting a synergistic antioxidant effect after 48h of treatment. As expected, H_2_O_2_ treatments served as a positive control and strongly elevated intracellular ROS.

The intracellular reduced glutathione (GSH) levels in OVCAR-3, OVCAR-8, and TOV-21G cell lines were measured after 48 h of incubation with the tested compounds, and the obtained values reflected changes in GSH levels as an indicator of the cellular antioxidant response (Figure 2C). Both LVC and VitC significantly increased GSH levels in OVCAR-8 cells (200% relative to control), whereas no significant changes were observed in OVCAR-3 and TOV-21G. A similar effect was maintained following treatment with the ATRi + VitC combination, particularly in OVCAR-8, where GSH levels reached around 250%. In OVCAR-3 and TOV-21G, GSH levels after ATRi + VitC treatment were also significantly higher compared with LVC or VitC alone. In OVCAR-3, only a moderate increase in GSH was observed after ATRi + VitC treatment, while in all other conditions, including ATRi alone and empty liposome treatment, GSH levels remained close to control values or showed a slight decrease.

### 2.3. ATR Inhibition Synergistically Interacts with LVC, Increasing Caspase-3-Independent Apoptotic Changes

**Figure 3:**
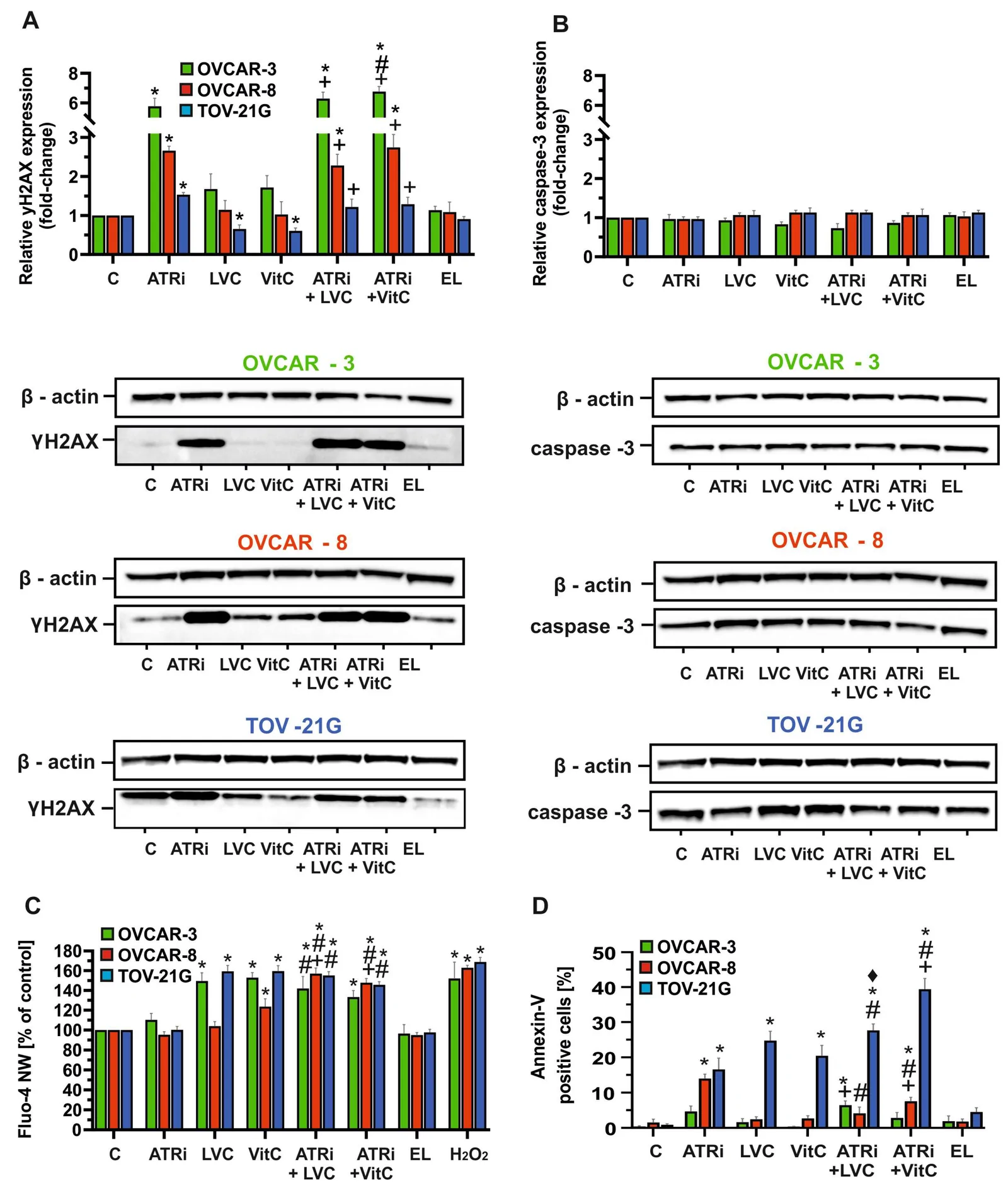
Analysis of apoptosis markers in ovarian cancer cell lines subjected to ATRi, LVC and VitC treatments. (**A**) Representative Western blot analysis of γH2AX. (**B**) Representative Western blot analysis of caspase-3. (**C**) Intracellular Ca^2+^ concentration measured after 48 h exposure. (**D**) Apoptosis was examined via dual staining with annexin V-FITC and PI, quantified with flow cytometry, and presented as a percentage of annexin V-positive (apoptotic) relative to untreated control cells. * Statistically significant differences between cells incubated with the compound compared with the control cells (*p* < 0.05). # Statistically significant changes between cells incubated with ATRi and combination treatment (ATRi + LVC; ATRi + VitC) (*p* < 0.05). + Statistically significant differences between the cells incubated with LVC or VitC and combination treatment (ATRi + LVC; ATRi + VitC) (*p* < 0.05). ♦ Statistically significant changes between combination treatments (ATRi + LVC and ATRi + VitC) (*p* < 0.05).

The effects of the tested compounds on DNA damage were assessed by analyzing the relative expression of the γH2AX histone via Western blot assay in OVCAR-3, OVCAR-8, and TOV-21G cell lines after 48 h of exposure to ATRi, LVC, VitC or their combinations. Treatment with ATRi alone markedly increased γH2AX levels in OVCAR-3 and OVCAR-8, suggesting enhanced DNA double-strand break formation. In TOV-21G, γH2AX expression after ATRi treatment was comparable to the control, indicating lower sensitivity to DNA damage induction. Neither free VitC nor LVC significantly altered γH2AX expression levels in any cell line. However, combination treatments of ATRi with VitC, regardless of formulation, led to a substantial increase in γH2AX in OVCAR-3 and OVCAR-8 compared with VitC alone, and a slight increase relative to ATRi alone, indicating a synergistic effect. In TOV-21G, increases in γH2AX were observed only under combination treatments, whereas LVC and VitC alone resulted in expression levels that were lower than the control (Figure 3A, Figure S4). Empty liposomes did not alter γH2AX levels in any cell line. Relative caspase-3 expression remained similar to control levels in all cell lines, regardless of treatment (Figure 3B).

Early apoptotic changes in the plasma membrane were evaluated using flow cytometry with Annexin V-FITC and propidium iodide staining after 48 h of treatment. The strongest increase in apoptosis was observed in TOV-21G after ATRi + LVC treatment, where apoptotic cells exceeded 27%. In OVCAR-3 and OVCAR-8, increases were smaller (~6% and ~7%, respectively). Both LVC and VitC increased apoptotic cell numbers, with the most pronounced effect for LVC in TOV-21G (~25%), while in OVCAR-3 and OVCAR-8 LVC-induced changes did not exceed 5%. Representative dot plots of annexin V-FITC- and PI-stained OC cells are presented in Figure S2.

Intracellular calcium (Ca^2+^) levels were measured using the Fluo-4 NW fluorescent probe. Free VitC significantly increased Ca^2+^ levels in all tested cell lines, whereas LVC induced elevated calcium only in OVCAR-3 and TOV-21G, Figure 3C. High Ca^2+^ levels were also observed following A + LVC and A + VitC treatments, suggesting a potential synergistic effect, supported by significant differences compared with single-agent treatments. ATRi alone did not produce significant changes in intracellular calcium levels relative to the control.

### 2.4. Oxidative and Ferroptotic Responses to ATR Inhibitor and Vitamin C Formulations in Ovarian Cancer Cell Lines

**Figure 4:**
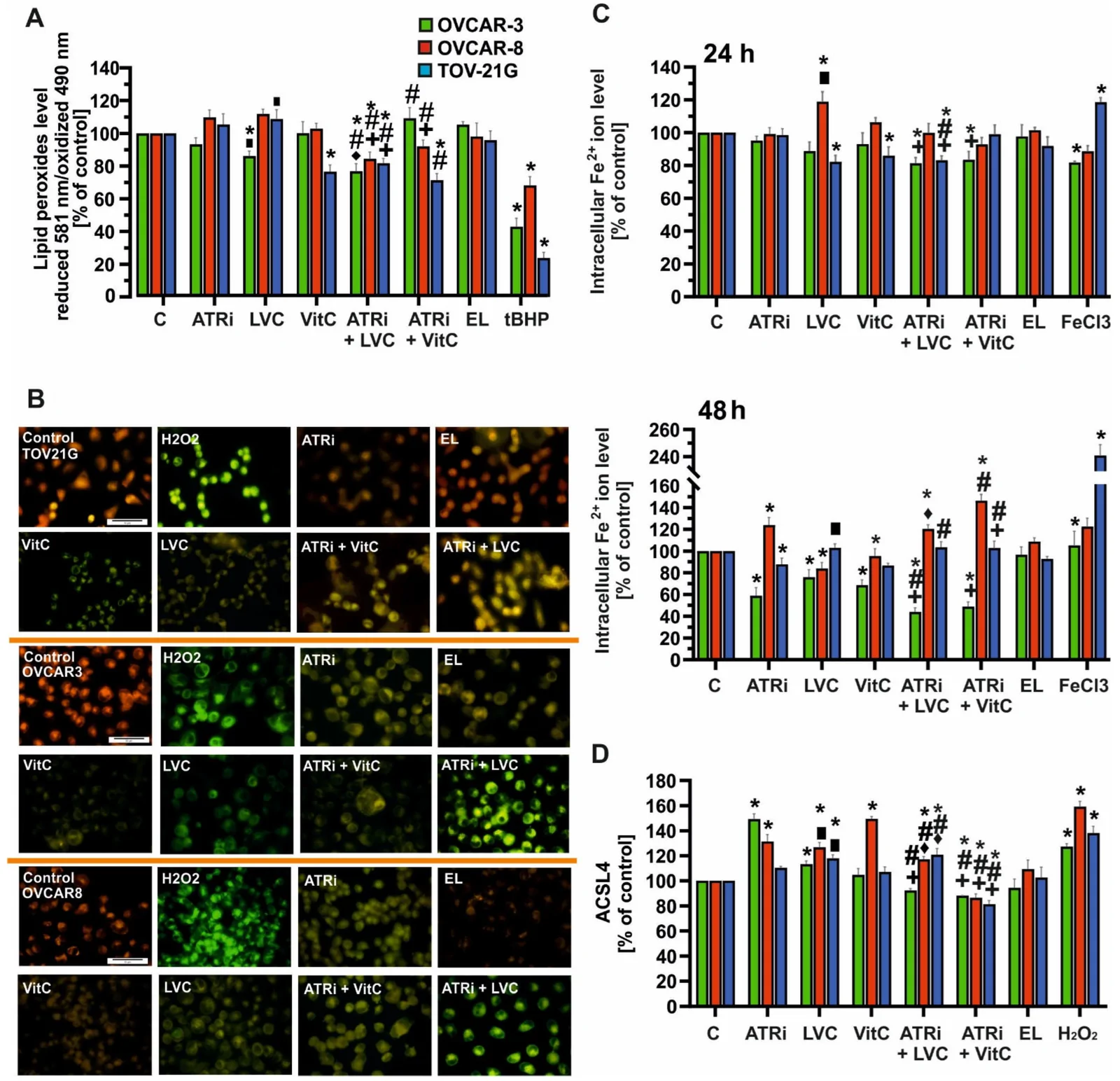
Effects of ATRi, liposomal vitamin C (LVC), and free vitamin C (VitC) on lipid peroxidation and iron metabolism in ovarian cancer cell lines. (**A**) Quantification of the C11-BODIPY (581/591) oxidation ratio after 48 h of treatment. (**B**) C11-BODIPY (581/591) imaging for lipid peroxidation, representative fluorescence images of OC cells treated with the tested compounds. Scale bar = 50 µm. (**C**) Intracellular Fe^2+^ levels measured after 24 h and 48 h of exposure. (**D**) ACSL4 expression levels after 48 h of treatment. * Statistically significant differences between cells incubated with the compound compared with the control cells (*p* < 0.05). # Statistically significant changes between cells incubated with ATRi and combination treatment (ATRi + LVC; ATRi + VitC) (*p* < 0.05). + Statistically significant differences between the cells incubated with LVC or VitC and combination treatment (ATRi + LVC; ATRi + VitC) (*p* < 0.05). ∎ Statistically significant differences between cells incubated with the LVC compared to VitC (*p* < 0.05). ♦ Statistically significant changes between combination treatments (ATRi + LVC and ATRi + VitC) (*p* < 0.05).

The level of lipid peroxidation (LPO) in OVCAR-3, OVCAR-8, and TOV-21G cell lines was assessed using the C11-BODIPY 581/591 fluorescent probe, which detects the peroxidation of unsaturated membrane phospholipids in response to oxidative stress. Cells were incubated for 48 h with the selected concentrations of compounds. Distinct responses were observed in all three cell lines (Figure 4A,B). In OVCAR-3 cells, a significant decrease in the fluorescence emission ratio, indicating increased lipid peroxidation, was observed after treatment with ATRi and LVC. Notably, only one combination, ATRi + LVC, enhanced LPO generation, while the ATRi + VitC combination did not increase LPO levels; instead, it significantly increased fluorescence ratios relative to ATRi alone, suggesting a protective antioxidant effect of vitamin C against oxidative stress. In OVCAR-8 cells, all treatments caused a significant increase in fluorescence ratios, reflecting a reduction in lipid peroxidation. Only the ATRi + VitC combination returned LPO levels close to control values, implying the strongest pro-oxidant effect among the treatments. Compared to OVCAR-3 and OVCAR-8, TOV-21G cells exhibited the highest tendency for LPO generation upon treatment. Significant decreases in fluorescence ratio, indicating enhanced lipid peroxidation, were recorded following all treatments except ATRi alone, which did not significantly affect LPO and remained similar to control levels. Empty liposomes did not significantly alter lipid peroxidation in any cell line.

Simultaneously, intracellular free iron ions (Fe^2+^) levels were measured after 24 and 48 h of incubation with the compounds. After 24 h, no significant changes in Fe^2+^ levels were observed after ATRi treatment in any cell line (Figure 4C). A notable increase in Fe^2+^ was seen only after LVC treatment in OVCAR-8 (120%), while no significant effects were found in OVCAR-3 or TOV-21G, with a decreasing trend observed in TOV-21G. Vitamin C treatment did not notably affect Fe^2+^ levels. Combinations of ATRi with LVC or VitC did not lead to significant Fe^2+^ accumulation; levels remained comparable to or slightly lower than the controls, particularly in OVCAR-3. After 48 h, the differences between cell lines became more pronounced. In OVCAR-3, all tested compounds reduced Fe^2+^ levels compared to the control, indicating depletion of the intracellular iron pool. Conversely, in OVCAR-8, nearly all treatments elevated Fe^2+^ levels, especially ATRi and ATRi + VitC. In TOV-21G cells, Fe^2+^ levels remained relatively stable across most treatments.

To evaluate ACSL4 expression levels, an ELISA was performed after 48 h of treatment (Figure 4D). Treatment with ATRi alone significantly increased ACSL4 levels in OVCAR-3 and OVCAR-8 cell lines, with the strongest effect observed in OVCAR-3. LVC and VitC also elevated ACSL4 expression, although to a lesser extent. Combination treatments (ATRi + LVC and ATRi + VitC) induced distinct responses depending on the cell line. In OVCAR-8 and TOV-21G, co-treatment with ATRi + VitC or ATRi + LVC resulted in a significant upregulation of ASCL4 compared with single-agent exposure. In contrast, OVCAR-3 cells demonstrated only moderate changes under combination treatment, remaining close to baseline control levels. Taken together, these findings indicate that ASCL4 expression is both treatment- and cell line-dependent, with the strongest induction observed under ATRi + VitC co-treatment in OVCAR-8 and TOV-21G cells.

## 3. Discussion

The combined effect of the ATRi, ceralasertib, and vitamin C, particularly in its liposomal form, has not yet been investigated in the context of ovarian cancer therapy. The combination of these two compounds may not only enhance the cytotoxic effect but could also influence the course of treatment in a manner dependent on the applied concentration of vitamin C. At lower doses, it may act protectively on normal cells, which, in clinical settings, translates into improved patient tolerance to treatment. The introduction of vitamin C as an adjuvant to anticancer therapy could make it possible to administer higher doses of the ATRi, thereby increasing its effectiveness while simultaneously reducing undesirable side effects. Thus, the proper selection of the vitamin C dose becomes crucial, as it may induce the desired therapeutic effect, potentially enhancing cancer cell death [11,12]. Importantly, its encapsulation in a liposomal carrier may increase bioavailability and facilitate the achievement of high concentrations at the target site, which would additionally and significantly potentiate its anticancer activity [14].

The first stage of the study involved 48 and 120 h incubations of OVCAR-3, OVCAR-8, and TOV-21G cells with selected drug concentrations in order to assess their cytotoxicity, both as monotherapies and in combination. Prior to this, the DT of each cell line was determined to align drug administration with the phase of rapid cellular proliferation. Among the analyzed cell lines, TOV-21G and OVCAR-8 exhibited the shortest DTs (22–27 h), consistent with previous reports, confirming the expected proliferation dynamics [20,21,22] and reflecting their highly proliferative and mesenchymal phenotypes [23,24]. Both cell lines harbor genetic alterations that promote cell cycle progression, including *ARID1A* deficiency, the mutation of KRAS (part of the RAS/MAPK pathway), *CCNE1* and *CCND1* overexpression, and the activation of PI3K/Akt signaling [25,26,27]. In contrast, OVCAR-3 displayed a substantially longer DT (≈49 h), in agreement with the literature data [28,29], which may be linked to *TP53* mutation, PIK3R1 mutation (regulatory subunit of phosphatidylinositol 3-kinase PI3K), and the high expression of epithelial markers such as keratin 18 and claudin 3, indicative of a more stable phenotype and reduced proliferation [30,31]. The variability in DT values across studies likely reflects differences in culture conditions, DT determination methods, and passage numbers.

Cytotoxicity analysis revealed that ATR inhibitors (Ceralasertib, ATRi) exhibited strong dose- and time-dependent effects in OVCAR-8 and TOV-21G cells, while OVCAR-3 showed marked resistance, maintaining ~45% viability after 48 h at 120 μM. Extended exposure (120 h) confirmed the high resistance of OVCAR-3, whereas TOV-21G remained the most sensitive. Similar cytotoxic activity of Ceralasertib has been demonstrated in breast cancer models, with IC_50_ values below 1 μM in most tested cell lines [32]. The differential response between ovarian lines may stem from their genetic background: OVCAR-3, derived from papillary adenocarcinoma, and OVCAR-8, originating from high-grade serous carcinoma, represent distinct molecular subtypes [33]. Both free (VitC) and liposomal (LVC) vitamin C formulations also exhibited time- and dose-dependent cytotoxicity. However, LVC exhibited stronger and, in some cases, faster cytotoxic activity compared to VitC, particularly after 48 h of incubation. The most pronounced differences were observed in OVCAR-8 and OVCAR-3 cells, which displayed higher resistance to ascorbate. In OVCAR-8 cells, following 48 h of treatment, LVC induced a significant reduction in cell viability at concentrations above 0.75 mM, with an IC_50_ of 1.73 mM. By comparison, VitC at the same time point had a weaker effect, with an IC_50_ of 1.87 mM, suggesting improved stability and cellular uptake. Similar findings with VitC were reported in other cancer models, including 4T1 breast cancer cells, in which VitC at concentrations of 0.6–1.1 mM markedly inhibited proliferation after 48–72 h of incubation [34]. Prolonging incubation to 120 h enhanced the activity of both formulations; however, the advantage of LVC over VitC remained most evident in OVCAR-3 and OVCAR-8 cells. The superior efficacy of LVC may be attributed to its increased stability, protection from oxidation, and enhanced cellular transport. This interpretation is supported by the literature reports and is consistent with studies indicating enhanced bioavailability and tumor retention of liposomal formulations [16]. Empty liposomes exerted minimal impact on cell viability, except at the highest concentrations and longest incubation times, where slight toxicity was observed. This effect may result from lipid accumulation causing osmotic stress or from stabilizing agents within the liposome that may exert toxicity and are consistent with earlier observations in other cancer models [35,36]. The combined treatment of ovarian cancer cell lines with ATR inhibitor (ATRi) and vitamin C formulations enhanced cytotoxicity compared with single-agent exposure. The strongest synergistic effects were observed in TOV-21G and OVCAR-8 cells. In contrast, OVCAR-3 cells exhibited pronounced resistance, with viability remaining above 67% after 48 h and a more evident decline observed only after 120 h. Vitamin C has frequently been investigated in combination with chemotherapeutic agents. For instance, one study demonstrated that VitC combined with paclitaxel or doxorubicin in breast cancer cell lines (MCF-7 and MDA-MB-231) markedly potentiated the cytotoxic effects of these drugs, showing particularly strong synergy with doxorubicin [37]. Another study reported that the intravenous administration of millimolar doses of VitC in combination with carboplatin and paclitaxel led to a synergistic cytotoxic effect in ovarian cancer cells, while also reducing treatment-related adverse effects in patients [12]. Overall, the data indicates that combining ATR inhibition with vitamin C, particularly in liposomal form, significantly augments cytotoxicity in ovarian cancer cells, with the magnitude of the response depending on cell line sensitivity and treatment duration.

An analysis of intracellular ROS levels revealed distinct redox responses across the studied ovarian cancer cell lines following treatment with the tested compounds. In TOV-21G cells, ROS levels were markedly lower than in OVCAR-8 and OVCAR-3, especially after a short incubation time. Our data are consistent with TOV-21G’s limited antioxidant capacity resulting from *ARID1A* mutations, which reduces SLC7A11 expression and GSH synthesis. Moreover, low GPx4 levels combined with high iron availability render these cells particularly susceptible to ferroptosis, even under conditions of moderate ROS levels [38,39]. This finding aligns with earlier reports showing that silencing *ARID1A* in wild-type cell lines, including TOV-21G, or restoring its function in mutant lines, correspondingly increased or reduced sensitivity to elesclomol, an ROS-generating agent [40]. In OVCAR-8 cells, short-term incubation led to a reduction in ROS, particularly after LVC treatment, and this remained consistent after prolonged exposure (48 h). At the same time, OVCAR-8 maintained relatively high viability, likely due to an efficient antioxidant system supported by GSH/GST activity and the Nrf2-mediated upregulation of GPx, catalase, and SOD [41,42]. OVCAR-3 cells showed a biphasic ROS response—the initial antioxidant activity of VitC and LVC was followed by ROS accumulation after 48 h. Although ROS levels after LVC or VitC treatment were the highest among all tested lines, OVCAR-3 retained strong viability, reflecting robust redox defense mechanisms. This resistance is associated with elevated GPx3 expression, conferring both intra- and extracellular protection, as well as high MnSOD activity [42,43,44]. These results correspond with previous findings in breast cancer cell lines, including MDA-MB-231 and MCF-7, demonstrating that pharmacological doses of ascorbate (doses of 0.5–20 mM for 24 h) can elevate ROS, induce DNA damage, and deplete ATP in cancer cells [45]. Importantly, the present data indicate that prolonged exposure to lower concentrations of VitC or LVC may similarly provoke oxidative stress, particularly in genetically predisposed ovarian cancer subtypes.

To further elucidate the oxidative balance in ovarian cancer cells, glutathione (GSH) levels were assessed to evaluate their contribution to antioxidant defense. The results confirmed that OVCAR-8 cells possess the most active GSH-dependent system [42]. In contrast, TOV-21G cells exhibited the lowest GSH activity due to the *ARID1A* mutation, which downregulates SLC7A11 expression and limits cystine uptake, leading to glutathione deficiency [38]. In OVCAR-3 cells, GSH levels remained relatively stable across treatments, suggesting a shift toward alternative antioxidant mechanisms, particularly GPx3-mediated detoxification, which functions both intra- and extracellularly [44,46]. A notable exception was the ATRi + LVC and VitC combination, which significantly increased GSH levels. A similar phenomenon was reported in breast cancer cell lines (MCF-7 and MDA-MB-231), where pharmacological doses of vitamin C (>0.5 mM) played a dual role: they induced oxidative stress (ROS increase), which in turn stimulated the GSH pathway to neutralize the generated H_2_O_2_ [47]. Although LVC demonstrated stronger cytotoxicity than VitC, it did not elicit a comparable rise in GSH levels, possibly due to the distinct cellular uptake mechanisms. Free VitC enters cells via SVCT1/2 transporters and promotes extracellular H_2_O_2_ formation, rapidly activating antioxidant defenses [48], whereas LVC is internalized mainly through endocytosis, leading to slower ascorbate release and more localized intracellular activity. Additionally, the limited active content within liposomes may contribute to this attenuated response [49].

Intracellular Ca^2+^ analysis revealed that all ovarian cancer cell lines exhibited elevated cytoplasmic calcium levels following treatment with VitC or LVC, either alone or combined with ATRi. This increase is a known early marker of apoptosis triggered by oxidative or mitochondrial stress [50]. The strongest calcium responses occurred in OVCAR-3 and TOV-21G cells, where concentrations exceeded 150% of the control values, indicating disrupted homeostasis and the activation of proapoptotic pathways. The ATRi + VitC combination produced the most pronounced effect, consistent with prior reports showing that pharmacological doses of vitamin C induce mitochondrial calcium overload and apoptosis in cancer cells [45,51]. Moreover, ATR prevents calcium-induced cell death, and an inhibitor would block this protective action [52]. Analysis of apoptosis-related markers revealed that elevated intracellular Ca^2+^ levels did not correspond with significant caspase activation. Caspase-3 expression remained near control levels across all treatments, indicating that cell death was largely caspase-independent. In contrast, γH2AX expression, reflecting DNA damage, was markedly increased, especially in OVCAR-3 cells treated with ATRi and its combinations with both forms of vitamin C, while OVCAR-8 and TOV-21G showed moderate and minimal induction. In SHIN3 ovarian cancer cells treated with sodium ascorbate (2–3.5 mM), significant activation of the DNA damage response pathway was detected within a few hours, in a time- and dose-dependent manner. This suggests that ascorbate, acting as a prooxidant, may induce double-strand breaks in DNA in cancer cells, as detected by γH2AX expression [12]. Flow cytometry further supported these observations. TOV-21G exhibited the highest proportion of apoptotic cells (~20%) following ATRi exposure, and the highest proportion when using ATRi with vitamin C or LVC at the same time (~39% and ~27%, respectively). Since Annexin V primarily detects phosphatidylserine externalization characteristic of apoptosis, the involvement of alternative cell death mechanisms such as caspase-independent apoptosis or ferroptosis cannot be excluded [53,54]. Similar observations were reported in MDAH 2774 breast cancer cells incubated for 1 h with 3 mM VitC, where cytometric analysis (Annexin V-FITC/PI) revealed no significant increase in Annexin V^+^ or PI^+^ cells, confirming a lack of classical apoptosis [55]. Overall, these results suggest that in the tested ovarian cancer cells, ATRi and LVC induce DNA damage and oxidative stress, leading to cell death.

Therefore, we focused our investigation on the ferroptosis pathway. Lipid peroxidation (LPO) provided further insight into ferroptosis-related processes potentially triggered by ascorbate. OVCAR-8 cells exhibited a higher red/green fluorescence ratio than controls, indicating lower lipid peroxidation compared to untreated cells. This suggests that OVCAR-8 effectively mitigates lipid damage, likely due to robust antioxidant systems [42]. However, the combined action of ATRi and the liposomal form of vitamin C led to lipid peroxidation. Conversely, TOV-21G showed a decreased ratio (except when treated with LVC), reflecting elevated lipid peroxidation and oxidative stress due to genetic mutations. OVCAR-3 displayed intermediate LPO levels, except after ATRi + LVC treatment, which induced maximal lipid peroxidation, whereas ATRi + VitC did not. In a study on anaplastic thyroid carcinoma (CAL-62 cells), pharmacological VitC doses (1–4 mM) for 24 h induced dose-dependent LPO, with maximal effects at 4 mM, indicating that vitamin C can target membranes and induce oxidative stress [56]. To further assess the involvement of ferroptosis, intracellular Fe^2+^ levels were measured. After 24 h of incubation, the greatest increases were observed in OVCAR-8 treated with LVC. After 48 h, the most pronounced Fe^2+^ increase occurred in OVCAR-8 treated with both combinations. This could enhance Fenton reactions and ROS generation, supporting the involvement of ferroptosis. Vitamin C has been shown to increase Fe^2+^ levels in pancreatic cancer cells by reducing Fe^3+^, promoting Fenton reactions and ferroptosis [57]. However, in some experimental groups, Fe^2+^ decreased compared to controls (e.g., OVCAR-3 and TOV-21G treated with VitC or its combination with ATRi), suggesting the activation of iron-buffering mechanisms such as ferritin storage or ferroportin-mediated export [8]. Alternatively, reduced Fe^2+^ availability may indicate minimal ferroptosis involvement, implying other death pathways, such as apoptosis or necroptosis.

In order to resolve the ambiguous results regarding the role of ferroptosis in ovarian cancer therapy using ceralsertib and new vitamin C nanoparticles, we investigated ACSL4 expression. ACSL4 facilitates the incorporation of polyunsaturated fatty acids into membrane phospholipids, promoting lipid peroxidation and ferroptotic cell death [58]. Its inhibition or loss has been shown to protect cells from ferroptosis-mediated damage [9,59]. ATRi treatment notably increased ACSL4 expression, particularly in OVCAR-3 and OVCAR-8 cells, while TOV-21G showed only moderate induction. Co-treatment with LVC further elevated ACSL4 levels in OVCAR-8 and TOV-21G, suggesting that oxidative stress combined with ATR inhibition enhances lipid remodeling pathways linked to ferroptosis. Conversely, OVCAR-3, characterized by strong redox buffering, showed minimal ACSL4 activation, which may underline its higher resistance. Overall, these findings indicate that ATRi and VitC/LVC co-treatment activates ACSL4-dependent ferroptotic pathways, particularly in redox-vulnerable ovarian cancer models such as TOV-21G and OVCAR-8, highlighting ferroptosis as a potential therapeutic target [60].

## 4. Materials and Methods

### 4.1. Materials

Ceralasertib (A, ATRi, AZD6738) was purchased from Wuhan ChemNorm Biotech (Wuhan, China). The inhibitor was dissolved in 100% dimethyl sulfoxide (DMSO), (Merck Life Science, Poznań, Poland) to create stock solutions, which were then stored at −80 °C for a maximum of 6 months. Liposomal vitamin C (LVC), potassium ascorbate (VitC), and empty liposomes (EL) were from Lipid Systems (Wroclaw, Poland). Cell culture reagents were purchased from Thermo Fisher Scientific (Waltham, MA, USA). Chemicals and solvents were purchased from Merck Life Science (Poznań, Poland) or Avantor Performance Materials Poland (Gliwice, Poland). Other key reagents used in the studies are included in Section 4 and Supplementary Table S1.

### 4.2. Cell Lines and Treatment

For the purpose of this study, three human ovarian cancer cell lines were used (OVCAR-3, OVCAR-8 and TOV-21G). OVCAR-3 and TOV-21G were acquired from the American Type Culture Collection (ATCC, Manassas, VA, USA) and OVCAR-8 cells were obtained from Institut de Recherche en Cancérologie de Montpellier, University of Montpellier, France. OVCAR-3 cells were cultivated in RPMI 1640 medium supplemented with 1.1% glucose and 15% fetal bovine serum (FBS), (Thermo Fisher Scientific, Waltham, MA, USA), OVCAR-8 cell line was cultivated in RPMI 1640 medium supplemented with 10% FBS, and TOV-21G in DMEM, 10% FBS in a cell culture incubator with an atmosphere of 5% CO_2_ at 37 °C and were routinely subcultured using 0.1% trypsin solution with 0.4 mM EDTA. During this study, the cells were thawed and passaged within 2 months of each experiment and regularly checked for mycoplasma contamination. The OVCAR-3 cells were treated with 2.5 μM ATRi (A), and 0.5 mM LVC, VitC or EL, OVCAR-8 cells were treated with 2.5 μM ATRi (A), and 0.75 mM LVC, VitC or EL, and TOV-21G cells were treated with 1 μM ATRi (A), and 0.25 mM LVC, VitC or EL during all experiments, chosen based on the CDI values. Experiments were performed with cells reaching a confluency of 80%. Unless stated otherwise, each experiment was independently repeated three times (n = 3).

### 4.3. Population Doubling Time

To determine the OVCAR-3, OVCAR-8 and TOV21G cell proliferation rates, we employed the trypan blue exclusion method [61]. Cells were seeded into 60 mm Petri dishes at a density of 2 × 10^5^ and then counted every 24 h. Briefly, 4% trypan blue solution was mixed with the cell suspension in a ratio of 1:1 and transferred to a Thoma chamber, and viable/nonviable cells were counted under an optical microscope. Based on the number of cells at the beginning and at each studied time point, we calculated the doubling time using the following formula DT = t/log2 (Nt/N0), where DT is the time required for the duplication of cell number, t is the time interval between the initial and final calculation of cell number, and N0 and Nt are the number of cells at the beginning and end of the experiment, respectively.

### 4.4. MTT Assay

Logarithmically growing cells (1.5 × 10^4^ for OVCAR-3 or 1 × 10^4^ for OVCAR-8 and TOV-21G for 48 h or 7 × 10^3^ for OVCAR-3 or 5 × 10^3^ for OVCAR-8 and TOV-21G for 120 h) were seeded into 96-well plates and, after 24 h, were treated with the indicated doses of ATRi (AZD6738), LVC, VitC and empty liposomes. After treatment, the medium in each well was aspirated, and the cells were washed twice with PBS, and incubated with 50 µL MTT (at a final concentration of 0.5 mg/mL) (Sigma Aldrich, St. Louis, MO, USA) for 4 h. Afterwards, 100 μL of DMSO was added to each well, the plates were incubated at room temperature or 37 °C protected from light until complete solubilization of the formazan crystals, and the samples were mixed for about 30 s using a plate shaker. The absorbance was determined spectrophotometrically on a microplate reader (Multiskan SkyHigh Microplate Spectrophotometer, Thermo Fisher Scientific, Waltham, MA, USA) at an experimental wavelength of 580 nm, using 720 nm as a reference wavelength. The coefficient of drug interaction (CDI) values were calculated using corrected absorbance values to determine the total cytotoxic effect of concurrent incubation with two inhibitors according to the formula: CDI = AB/A × B [62]. Based on the corrected absorbance of each group, AB is the cell viability of the two-agent combination group, and each A or B is the cell viability of the single-agent group. CDI values indicated whether the effect exerted by the combination of drugs was strongly synergistic (CDI  <  0.30), synergistic (0.3  ≤  CDI  <  0.7), moderately synergistic (0.7  ≤  CDI  <  0.85), slightly synergistic (0.85  ≤  CDI  <  1.0), additive (CDI  =  1.00) or antagonistic (CDI  >  1.00).

### 4.5. Intracellular ROS Generation

The intracellular generation of ROS was measured using a DCFH2-DA probe (Thermo Fisher Scientific, Waltham, MA, USA). Intracellular ROS levels following short-term exposure to the compounds were determined directly in cell monolayers in 96-well microplates using a Fluoroskan Ascent FL microplate reader (Labsystems, Stockholm, Sweden). Cells were preincubated with DCFH2-DA in the culture medium without phenol red at a final concentration of 5 µM for 30 min at 37 °C. The kinetics of ROS generation in the cells after treatment with corresponding doses of compounds were measured for up to 210 min. A total of 100 µM of H_2_O_2_ was used as positive control. The fluorescence of DCF was measured at ~530 nm after excitation at ~485 nm (DCFH2-DA, after deacetylation to DCFH2, is oxidized intracellularly to its fluorescent derivative, DCF). Similarly, for long-term exposure to the respective compounds, the cells treated with the drugs were incubated for 48 h (37 °C, 5% CO_2_). After the incubation period, the medium was removed, and each well was washed three times with PBS. Next, 5 μM of the probe was added to each well, followed by incubation for 60 min (37 °C, 5% CO_2_), after which the fluorescence was measured. To minimize errors in fluorescence intensity measurements resulting from potential cell detachment and subsequent reduction in cell number due to drug treatment, DNA content was also quantified. After measuring ROS production, the probes were centrifuged at 300× *g* for 10 min. The cell monolayers were then washed three times with PBS, and the plate was frozen at −70 °C. Immediately prior to further measurements, the microplate was thawed at room temperature, after which 100 μL of deionized water was added to the appropriate wells, and the plate was refrozen at −70 °C. Following a second thawing, RNA was digested using 50 μL of RNase at a concentration of 10 μg/mL per well. Subsequently, 100 μL of 5 μM propidium iodide (PI) was added to each well. The plate was immediately shaken and incubated for 15 min at room temperature in the dark. Fluorescence was then measured at an excitation/emission wavelength of 350/620 nm using a Fluoroskan Ascent FL microplate reader (Labsystems, Stockholm, Sweden).

### 4.6. Intracellular Calcium Assay

Intracellular calcium levels were assessed using the fluorescent probe Fluo-4 NW (Fluo-4 NW Calcium Assay Kit, Molecular Probes, Eugene, OR, USA), Table S2. Cells were seeded in black 96-well plates at a density of 1.5 × 10^4^ (OVCAR-3) and of 1 × 10^4^ (OVCAR-8 and TOV-21G) cells per well and treated for 48 h with corresponding concentrations of the compounds. Following treatment, the medium was removed, and the cells were washed with PBS. Subsequently, a dye loading solution (containing Fluo-4 NW dye, probenecid, and assay buffer composed of 1× HBSS and 20 mM HEPES; 100 μL per well) was prepared according to the manufacturer’s instructions. The plates were incubated in the dark for 30 min at 37 °C, followed by an additional 30 min incubation at room temperature. Fluorescence was measured using a microplate reader at an excitation wavelength of ~494 nm and an emission wavelength of ~516 nm. To minimize errors in fluorescence intensity measurements due to potential cell detachment and subsequent reduction in cell number caused by drug treatment, DNA content was quantified in the same manner as in the ROS generation assay.

### 4.7. Phosphatidylserine Externalization Measurement

The double staining of cells with annexin V and propidium iodide was used to assess the type of cell death. The visualization of cells stained with annexin V-Alexa Fluor™ 488 and PI was applied according to the protocol of the manufacturer (cat: V13245, Invitrogen, Thermo Fisher Scientific, Waltham, MA, USA). Briefly, 2 × 10^5^ control and drug-treated cells seeded onto 60 mm Petri dishes after 48 h were washed with cold PBS and resuspended in 140 μL binding buffer (delivered from the producer) that contained 3 μL of annexin V-Alexa Fluor™ 488, 1 μL 100 μg/mL of PI and stained for 15 min on ice. Finally, at least 10^4^ cells were analyzed for Alexa Fluor™ 488 and PI fluorescence (Ex ~488 nm; Em ~530 nm) using a flow cytometer (LSR II, Becton Dickinson, Franklin Lakes, NJ, USA). With the use of the annexin V-Alexa Fluor™ 488 and propidium iodide (PI) double-staining regime, four populations of cells were distinguishable in a two-color flow cytometry: normal cells: annexin V-Alexa Fluor™ 488-negative, PI-negative; early apoptotic cells: annexin V-Alexa Fluor™ 488-positive, PI-negative; late apoptotic cells: annexin V-Alexa Fluor™ 488-positive and PI-positive; dead cells: annexin V-Alexa Fluor™ 488-negative and PI-positive. The populations of cells were quantified from a standard count of 10^4^ cells using FlowJo software v7.6 (Ashland, OR, USA).

### 4.8. Lipid Peroxidation Assay

Lipid peroxidation was assessed using the fluorescent probe C11-BODIPY 581/591 (cat. SML3717, Sigma-Aldrich, Merck; Darmstadt, Germany). Cells were seeded in black 96-well plates at a density of 1.5 × 10^4^ (OVCAR-3) and of 1 × 10^4^ (OVCAR-8 and TOV-21G) cells per well and treated for 48 h with corresponding concentrations of the compounds. Following treatment, the medium was removed, and the cells were washed with PBS. Subsequently, 100 μL of the probe solution, prepared at a final concentration of 5 μM in accordance with the manufacturer’s instructions, was added to each well. The cells were then incubated for 30 min at 37 °C in a 5% CO_2_ incubator. After incubation, the cells were washed twice with 100 μL of PBS, and fluorescence was measured using a Fluoroskan Ascent FL microplate reader (Labsystems, Helsinki, Finland). Emission was detected at ~610 nm upon excitation at ~581 nm for the reduced (non-oxidized) form of the probe, and at ~530 nm emission following ~490 nm excitation for the oxidized form. To quantify the extent of lipid peroxidation, the ratio of fluorescence intensities between the reduced and oxidized forms was calculated. The result was expressed as the percentage of lipid peroxidation according to the following equation: LPO = fluorescence of the reduced form/fluorescence of the oxidized form × 100%. Treatment with 1 mM tert-butyl hydroperoxide (tBHP) for 30 min was used as the positive control. To minimize errors in fluorescence intensity measurements due to potential cell detachment and subsequent reduction in cell number caused by drug treatment, DNA content was quantified in the same manner as in the ROS generation assay.

### 4.9. Cell Ferrous Iron Measurement

For intracellular iron detection, a cell ferrous iron (Fe^2+^) fluorometric assay kit (cat. E-BC-F101; Elabscience Biotechnology Inc.; Houston, TX, USA) was used, following the manufacturer’s instructions. Briefly, cells were seeded in black 96-well plates at a density of 1.5 × 10^4^ (OVCAR-3) and of 1 × 10^4^ (OVCAR-8 and TOV-21G) cells per well and treated for 24 or 48 h with corresponding concentrations of the compounds. After treatment, the medium was removed, and each well was washed with 100 μL of the 1:9 diluted buffer working solution provided with the kit. Next, 50 μL of the fluorescent probe, prepared at a final concentration of 5 μM according to the manufacturer’s instructions, was added to each well. The plates were incubated for 45 min at 37 °C in 5% CO_2_ and protected from light. After incubation, cells were washed twice with 100 μL of the working buffer solution, and fluorescence was measured at 542 nm excitation and 575 nm emission using a Fluoroskan Ascent FL microplate reader (Labsystems, Helsinki, Finland). Ferric chloride (FeCl_3_) was used as a positive control at a final concentration of 100 μM.

### 4.10. Western Blot Analysis

The cells were seeded in 100 mm dishes (1.5 × 10^6^ cells) and then treated for 48 h in order to analyze protein expression levels. Following the treatment, the whole-cell lysates were prepared in ice-cold RIPA buffer (Thermo Fisher Scientific, Waltham, MA, USA) supplemented with phenylmethylsulfonyl fluoride (1 mM PMSF, Merck Life Science (Poznań, Poland)), Halt Protease Inhibitor Cocktail (Thermo Fisher Scientific), and Halt Phosphatase Inhibitor Cocktail (Thermo Fisher Scientific). Then, the samples were sonicated and centrifuged, and mPAGE 4X LDS Sample Buffer supplemented with 50 mM β-mercaptoethanol (Merck Life Science (Poznań, Poland)) was used as a sample buffer. A total of 30 μg of proteins was loaded into each lane of the mPAGE Bis-Tris gels, and then, the proteins were separated by SDS polyacrylamide gel electrophoresis at 180 V for 30 min in an electrophoresis tank (Mini-PROTEAN Tetra Cell, Bio-Rad, Hercules, CA, USA) using MOPS SDS running buffer, Merck Life Science (Poznań, Poland). Next, the proteins were transferred onto 0.45 µm PVDF membranes using a semi-dry transfer Trans-Blot Turbo Transfer System (Bio-Rad, Hercules, CA, USA) in mPAGE Transfer Buffer according to the mPAGE Bis-Tris gel manufacturer’s optimized instructions. After blocking nonspecific sites with 5% non-fat dry milk in TBST for 1 h and washing with TBST, the membranes were incubated overnight at 4 °C with rabbit monoclonal antibodies (1:1000) against γH2AX (cat. no.: 9718), and caspase-3 (cat. no.: D3R6Y), from Cell Signaling Technology, Inc. (Danvers, MA, USA), and mouse monoclonal anti-β-actin antibody (cat. no. A1878, Merck Life Science (Poznań, Poland)), followed by incubation with anti-rabbit IgG horseradish peroxidase-conjugated (cat. no.: 7074, Cell Signaling Technology) or anti-mouse IgG HRP-conjugated (cat. no.: A28177, Invitrogen, Thermo Fisher Scientific, Waltham, MA, USA) secondary antibodies for 1 h at room temperature, Table S3. To obtain a chemiluminescence signal, an enhanced chemiluminescent (ECL) substrate was used (SuperSignal™ West Pico PLUS Chemiluminescent Substrate (Thermo Fisher Scientific, Waltham, MA, USA)). Images were visualized using a c300 Azure imaging system (Azure Biosystems, Dublin, CA, USA). Densitometric quantification of the immunoreactive bands was performed with ImageJ software v1.5. Relative protein levels were expressed as the ratio of the densitometric volume of the tested band to that of the respective β-actin band.

### 4.11. Quantification of Reduced Glutathione (GSH) Levels

A reduced glutathione (GSH) colorimetric assay Kit (cat. E-BC-K030-M; Elabscience Biotechnology Inc., Houston, TX, USA) was used to measure intracellular GSH levels. Prior to quantitative analysis of reduced glutathione (GSH) in cell lysates, prepared as described above for the Western blot method, a standard curve was generated using GSH standards ranging from 0 to 100 μmol/L, following the manufacturer’s instructions and using the reagents provided with the assay kit (Figure S3A). From the prepared cell lysates, 100 μL of aliquots was transferred into new Eppendorf tubes. Subsequently, 100 μL of acid reagent was added to each sample and thoroughly mixed. Samples were centrifuged at 4 °C for 10 min at 4500× *g*. After centrifugation, 100 μL of the resulting supernatant was transferred to a 96-well microplate. To each well containing the test sample, 25 μL of 5,5′-dithiobis-(2-nitrobenzoic acid) (DTNB) reagent was added. For blank wells, 100 μL of acid reagent was added, while wells for the standard curve contained 100 μL of previously prepared GSH standard solutions at varying concentrations. Then, 100 μL of phosphate buffer was added to all wells, followed by thorough mixing. The plate was incubated for 5 min at room temperature in the dark. Absorbance was measured at 405 nm using a Multiskan SkyHigh Microplate Spectrophotometer (Thermo Fisher Scientific, Waltham, MA, USA).

### 4.12. Enzyme-Linked Immunosorbent Assay (ELISA) for ACSL4

The expression levels of ACSL4 protein in cell lysates were determined using the ACSL4 ELISA Kit (cat. ELK8993, ELK Biotechnology Co., Ltd., Wuhan, China) according to the manufacturer’s instructions. Briefly, cell lysates were prepared and diluted appropriately in the provided sample diluent. Then, 100 μL of each sample or standard was added to the pre-coated 96-well microplate and incubated for 1 h at 37 °C to allow specific binding of ACSL4 to the immobilized antibodies. Following incubation, wells were washed three times with the washing buffer to remove unbound material. Next, 100 μL of biotinylated detection antibody specific for ACSL4 was added to each well and incubated for 50 min at 37 °C. After washing, 100 μL of horseradish peroxidase (HRP)-streptavidin conjugate was added and incubated for 50 min at 37 °C in the dark. The plate was washed again to remove excess conjugate before adding 90 μL of 3,3′,5,5′-Tetramethylbenzidine (TMB) substrate solution to each well. The enzymatic reaction was allowed to proceed for 20 min at room temperature in the dark. The reaction was stopped by adding 50 μL of stop solution, changing the color from blue to yellow. Absorbance was measured immediately at 450 nm using a Multiskan SkyHigh Microplate Spectrophotometer (Thermo Fisher Scientific, Waltham, MA, USA). The ACSL4 concentration in each sample was calculated from a standard curve generated using the supplied ACSL4 standards (Figure S3B).

### 4.13. Statistical Analysis

Statistical analysis was performed with Statistica version 13.1 (StatSoft, Tulsa, OK, USA). The normality of data distribution was evaluated using the Shapiro–Wilk test. The homogeneity of variance within groups was assessed using the Brown–Forsythe test. The statistical significance of differences among multiple groups was determined using ordinary one-way or two-way ANOVA followed by Tukey’s multiple comparison tests. The data are presented as the mean ± SD of at least three independent experiments. *p* values of less than 0.05 were considered statistically significant.

## 5. Conclusions

**Figure 5:**
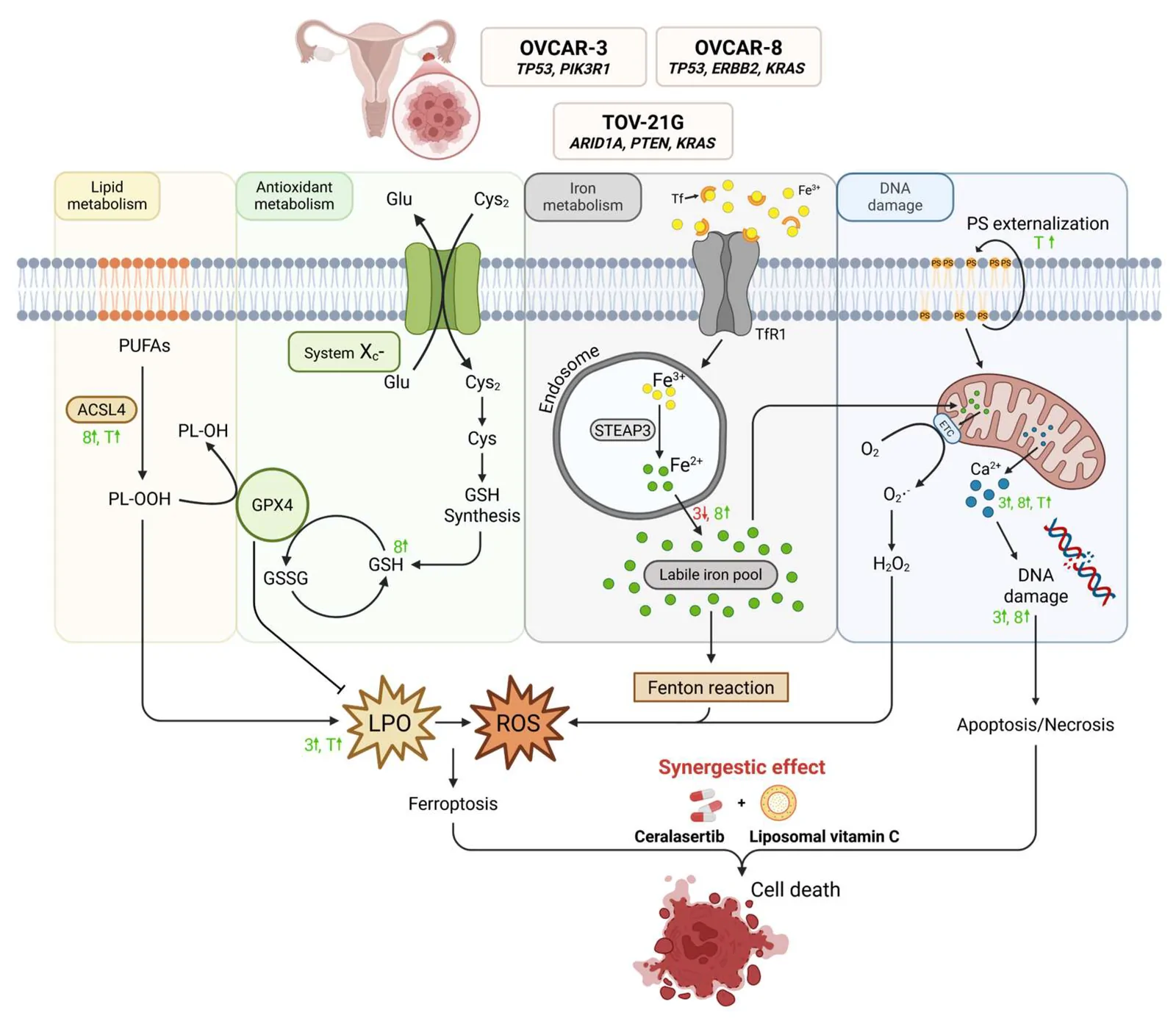
Molecular mechanism of sensitizing ovarian cancer cells to ceralasertib after the administration of liposomal vitamin C (LVC) in 3 (OVCAR-3), 8 (OVCAR-8), and T (TOV-21G) cells. Created in BioRender. Rogalska, A. (2026) https://BioRender.com/k0hwwwm, accessed on 28 January 2025. Together, the drugs act synergistically and cause several types of death, such as ferroptosis or apoptotic–necrotic changes. Ferroptosis can be induced by lipid (1), antioxidant (2) or iron (3) metabolism. (1) Under conditions of oxidative stress, free PUFAs are catalyzed by ACSL4, leading to the formation of toxic PL-OOH. (2) Antioxidant defense metabolism. Extracellular cystine is exchanged with glutamate to enter the cell through system Xc^−^, and it converts into cysteine to promote GSH synthesis. GPX4 transforms GSH into GSSG and simultaneously reduces toxic lipid hydroperoxides (LOOHs). The combined administration of ATRi and LVC can trigger ferroptosis by promoting lipid-ROS production. (3) Iron metabolism. Extracellular Fe^3+^ enters the cell through transferrin and TfR1. In cells, Fe^3+^ is released from transferrin and reduced to Fe^2+^ by STEAP3 and forms a labile iron pool in the cell412. Fe^2+^ contributes to the formation of ROS and oxidative stress through the Fenton reaction. These reactions initiate the peroxidation of cellular macromolecules, including lipids, nucleic acids, and proteins, driving ferroptosis. (4) DNA damage. The combined action of ATRi and LVC leads to DNA damage by inhibiting repair capabilities in the cell cycle, calcium efflux, and oxidative stress, which leads to cell death. ACSL4, acyl-CoA synthetase long-chain family member 4; ERBB2, receptor tyrosine–protein kinase, PUFA, polyunsaturated fatty acid; GPX4, glutathione peroxidase 4; GSH, glutathione; GSSG, glutathione disulfide; PTEN (phosphatase and tensin homolog, tumor suppressor); ROS, reactive oxygen species; STEAP3, six-transmembrane epithelial antigen of prostate 3; TfR1, transferrin receptor 1.

This study demonstrates, for the first time, the potential of combining the ATR inhibitor ceralasertib (ATRi) with vitamin C, particularly in its liposomal form (LVC), in the treatment of ovarian cancer (Figure 5). The results indicate that both agents exert dose- and time-dependent cytotoxic effects, which are strongly enhanced when administered together. The magnitude of response depended on the genetic and metabolic characteristics of each ovarian cancer cell line, with *ARID1A*-mutated TOV-21G and highly proliferative OVCAR-8 cells showing the greatest sensitivity, while OVCAR-3 cells remained relatively resistant. Mechanistically, the combined treatment induced peroxide stress, elevated intracellular calcium, and DNA damage, predominantly leading to non-apoptotic, caspase-independent cell death. Evidence from lipid peroxidation and Fe^2+^ analyses suggest the involvement of ferroptosis, particularly in redox-vulnerable lines. The upregulation of ACSL4 expression following ATRi and LVC co-treatment further supports the activation of ferroptotic pathways, consistent with previous reports linking ACSL4 to lipid remodeling and sensitivity to ferroptosis. Liposomal encapsulation of vitamin C enhanced its cytotoxic efficacy compared to free ascorbate, likely by improving intracellular delivery and bioavailability. This formulation-dependent effect underscores the importance of optimizing vitamin C concentration and delivery systems in potential clinical applications. At pharmacologically achievable doses, vitamin C may act not only as a cytotoxic cofactor but also as a modulator of redox balance, potentially enhancing ATR inhibitor efficacy while limiting systemic toxicity. These findings highlight the therapeutic promise of combining ATR pathway inhibition with oxidative modulation by liposomal vitamin C as a novel strategy to enhance ovarian cancer cell death through ferroptosis-related mechanisms.

## Figures and Tables

**Table 1:** IC50 (±standard deviation (SD)) values of different cell lines—cytotoxicity assays.

		IC50 (µM) + SD	
Cell Line		48 h	120 h
**OVCAR-3**	**ATR**	26.2 ± 1.88	0.87 ± 0.34
	**VitC**	1.92 ± 0.05	0.69 ± 0.21
	**LVC**	1.29 ± 0.1	0.72 ± 0.24
**OVCAR-8**	**ATR**	4.38 ± 0.5	1.85 ± 0.25
	**VitC**	1.87 ± 0.52	0.72 ± 0.29
	**LVC**	1.73 ± 0.44	0.61 ± 0.24
**TOV-21G**	**ATR**	3.98 ± 0.93	1.47 ± 0.15
	**VitC**	0.29 ± 0.08	0.15 ± 0.13
	**LVC**	0.26 ± 0.08	0.19 ± 0.12

## Data Availability

The original contributions presented in this study are included in the article and Supplementary Materials. Further inquiries can be directed to the corresponding author.

## Supplementary Materials

The following supporting information can be downloaded at: https://www.mdpi.com/article/10.3390/ijms27062630/s1.

## Abbreviations

The following abbreviations are used in this manuscript:

A	ATR inhibitor (ceralasertib)
ATR	Ataxia Telangiectasia and RAD3-related protein
ACSL4	Long-Chain-fatty-Acid—CoA ligase
ATRi	ATR inhibitor(s)
C	Vitamin C
CDI	Coefficient of Drug Interaction
DSB	Double-Strand Break
FBS	Fetal Bovine Serum
GSH	Reduced Glutathione
HR	Homologous Recombination
LVC	Liposomal Vitamin C
NHEJ	Non-Homologous End Joining
OC	Ovarian Cancer
PARP1	PARP inhibitor(s)
SSB	Single-Strand Break
