# Correction: Gralewska-Zając et al. Liposomal Vitamin C as a Modulator of the Efficacy of Ceralasertib Therapy in Ovarian Cancer. *Int. J. Mol. Sci.* 2026, *27*, 2630

**DOI:** 10.3390/ijms27156987

**Published:** 2026-08-04

**Authors:** Patrycja Gralewska-Zając, Aleksandra Przybylska, Marek Langner, Magdalena Przybyło, Agnieszka Marczak, Aneta Rogalska

**Affiliations:** 1 Department of Medical Biophysics, Institute of Biophysics, Faculty of Biology and Environmental Protection, University of Lodz, 141/143 Pomorska Street, 90-236 Lodz, Poland; patrycja.gralewska.zajac@biol.uni.lodz.pl (P.G.-Z.); aleksandra.przybylska@edu.uni.lodz.pl (A.P.); agnieszka.marczak@biol.uni.lodz.pl (A.M.); 2 Department of Biomedical Engineering, Wroclaw University of Science and Technology, 13 Grunwaldzki Square, 50-377 Wrocław, Poland; marek.langner@lipid-systems.pl (M.L.); magdalena.przybylo@lipid-systems.pl (M.P.); 3 Lipid Systems Ltd., 48C Krzemieniecka Street, 54-613 Wrocław, Poland

In the original publication [1], there was a mistake in Figure 4B—two pairs of images were identical (OVCAR3 Control and OVCAR8 LVC; OVCAR3 EL and OVCAR8 EL). In the corrected Figure 4B, the OVCAR8 LVC and OVCAR3 EL images have been replaced with the correct ones. The authors state that the scientific conclusions are unaffected. This correction was approved by the Academic Editor. The original publication has also been updated.

## Figures and Tables

**Figure 4:**
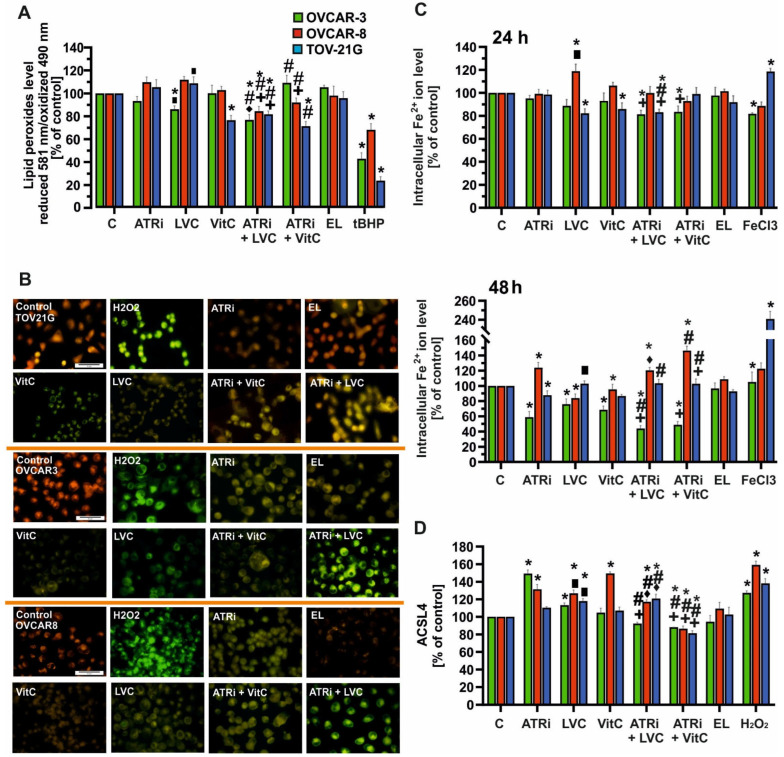
Effects of ATRi, liposomal vitamin C (LVC), and free vitamin C (VitC) on lipid peroxidation and iron metabolism in ovarian cancer cell lines. (**A**) Quantification of the C11-BODIPY (581/591) oxidation ratio after 48 h of treatment. (**B**) C11-BODIPY (581/591) imaging for lipid peroxidation, representative fluorescence images of OC cells treated with the tested compounds. Scale bar = 50 µm. (**C**) Intracellular Fe^2+^ levels measured after 24 h and 48 h of exposure. (**D**) ACSL4 expression levels after 48 h of treatment. * Statistically significant differences between cells incubated with the compound compared with the control cells (*p* < 0.05). # Statistically significant changes between cells incubated with ATRi and combination treatment (ATRi + LVC; ATRi + VitC) (*p* < 0.05). + Statistically significant differences between the cells incubated with LVC or VitC and combination treatment (ATRi + LVC; ATRi + VitC) (*p* < 0.05). ∎ Statistically significant differences between cells incubated with the LVC compared to VitC (*p* < 0.05). ♦ Statistically significant changes between combination treatments (ATRi + LVC and ATRi + VitC) (*p* < 0.05).
