# Correction: Gralewska-Zając et al. Liposomal Vitamin C as a Modulator of the Efficacy of Ceralasertib Therapy in Ovarian Cancer. *Int. J. Mol. Sci.* 2026, *27*, 2630

**DOI:** 10.3390/ijms27219440

**Published:** 2026-10-23

**Authors:** Patrycja Gralewska-Zając, Aleksandra Przybylska, Marek Langner, Magdalena Przybyło, Agnieszka Marczak, Aneta Rogalska

**Affiliations:** 1 Department of Medical Biophysics, Institute of Biophysics, Faculty of Biology and Environmental Protection, University of Lodz, 141/143 Pomorska Street, 90-236 Lodz, Poland; patrycja.gralewska.zajac@biol.uni.lodz.pl (P.G.-Z.); aleksandra.przybylska@edu.uni.lodz.pl (A.P.); agnieszka.marczak@biol.uni.lodz.pl (A.M.); 2 Department of Biomedical Engineering, Wroclaw University of Science and Technology, 13 Grunwaldzki Square, 50-377 Wrocław, Poland; marek.langner@lipid-systems.pl (M.L.); magdalena.przybylo@lipid-systems.pl (M.P.); 3 Lipid Systems Ltd., 48C Krzemieniecka Street, 54-613 Wrocław, Poland

**Error in Figure**

In the original publication [1], there was a mistake in Figure 3A,B. The β-actin loading control panel for TOV-21G cells was selected from a parallel experimental replicate rather than the corresponding target protein blot shown in the figure. The original β-actin data from the correct replicate in TOV-21G cells have been provided in the corrected version of Figure 3A,B and in Figure S4. The corrected legend of Figure 3B appears below.

In Figure S4, the same β-actin loading control was presented for histone and caspase analyses in OVCAR-8 cells because both target proteins were derived from the same membrane in experimental replicate 1. For experimental replicates 2 and 3, the corresponding β-actin loading controls for histone analysis were included. The legend for Figure S4 has also been clarified to provide additional information regarding the β-actin loading controls.

**Text Correction**

A correction has been made to the text relevant to Figure 3 by adding a clarification to Materials and Methods, Section 4.10. Western Blot Analysis, after the sentence:

“Transfer System (Bio-Rad, Hercules, CA, USA) in mPAGE Transfer Buffer according to the mPAGE Bis-Tris gel manufacturer’s optimized instructions.”

The following sentence has been added:

“Where appropriate, membranes were cut according to the molecular weight of the target proteins after transfer, and individual membrane sections were incubated separately with the respective primary antibodies.”

The authors state that the interpretation of the results and scientific conclusions are unaffected by the revision. These corrections were approved by the Academic Editor. The original publication has also been updated.

## Figures and Tables

**Figure 3:**
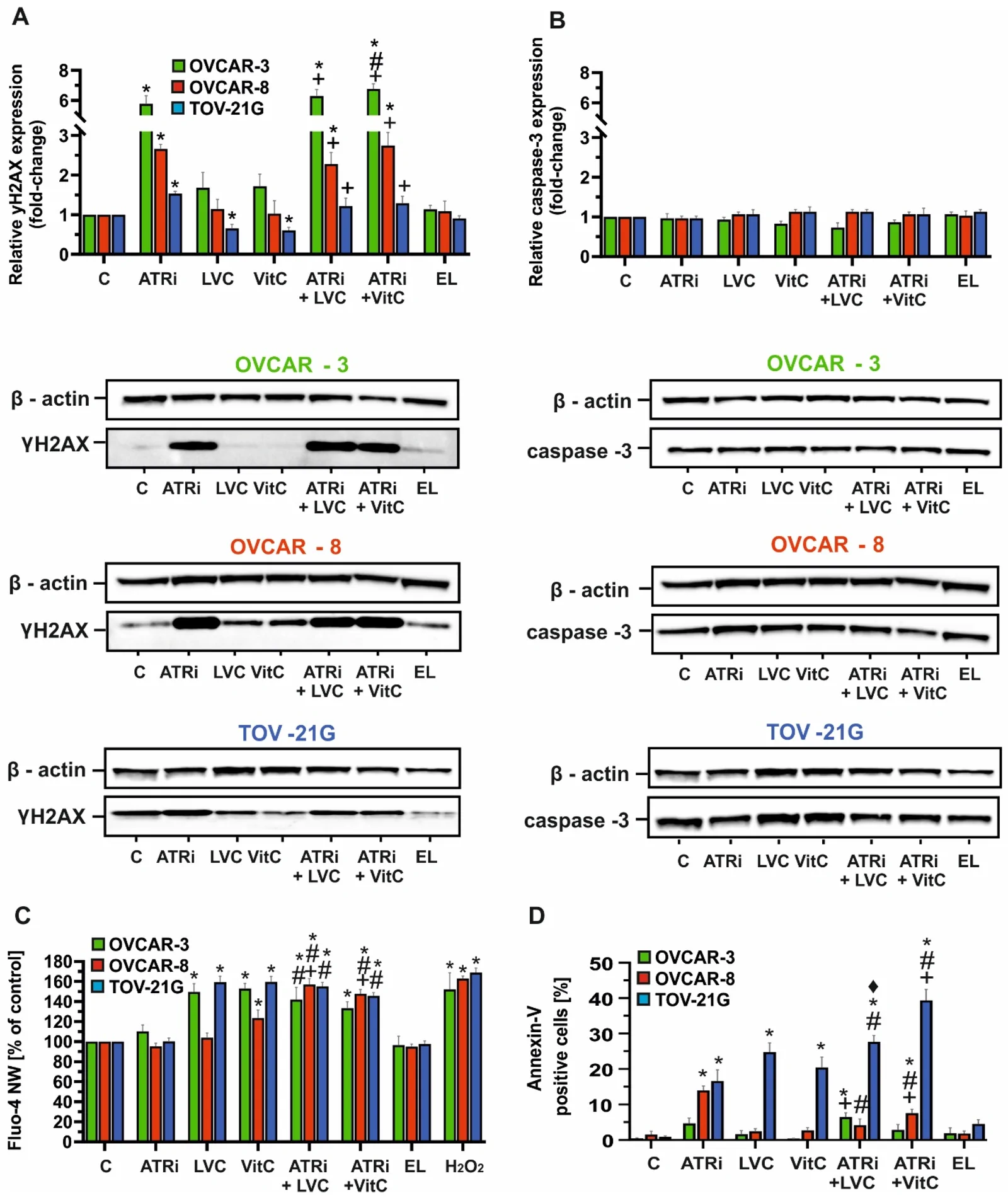
Analysis of apoptosis markers in ovarian cancer cell lines subjected to ATRi, LVC and VitC treatments. (**A**) Representative Western blot analysis of γH2AX. (**B**) Representative Western blot analysis of caspase-3. The same β-actin loading control is shown for caspase-3 and γH2AX in OVCAR-8 and TOV-21G cells because both proteins were analysed on separate sections of the same membrane prepared from the same protein samples. (**C**) Intracellular Ca^2+^ concentration measured after 48 h exposure. (**D**) Apoptosis was examined via dual staining with annexin V-FITC and PI, quantified with flow cytometry, and presented as a percentage of annexin V-positive (apoptotic) cells relative to untreated control cells. * Statistically significant differences between cells incubated with the compound compared with the control cells (*p* < 0.05). # Statistically significant changes between cells incubated with ATRi and cells receiving the combination treatment (ATRi + LVC; ATRi + VitC) (*p* < 0.05). + Statistically significant differences between the cells incubated with LVC or VitC and combination treatment (ATRi + LVC; ATRi + VitC) (*p* < 0.05). ♦ Statistically significant changes between combination treatments (ATRi + LVC and ATRi + VitC) (*p* < 0.05).

**Figure S4:**
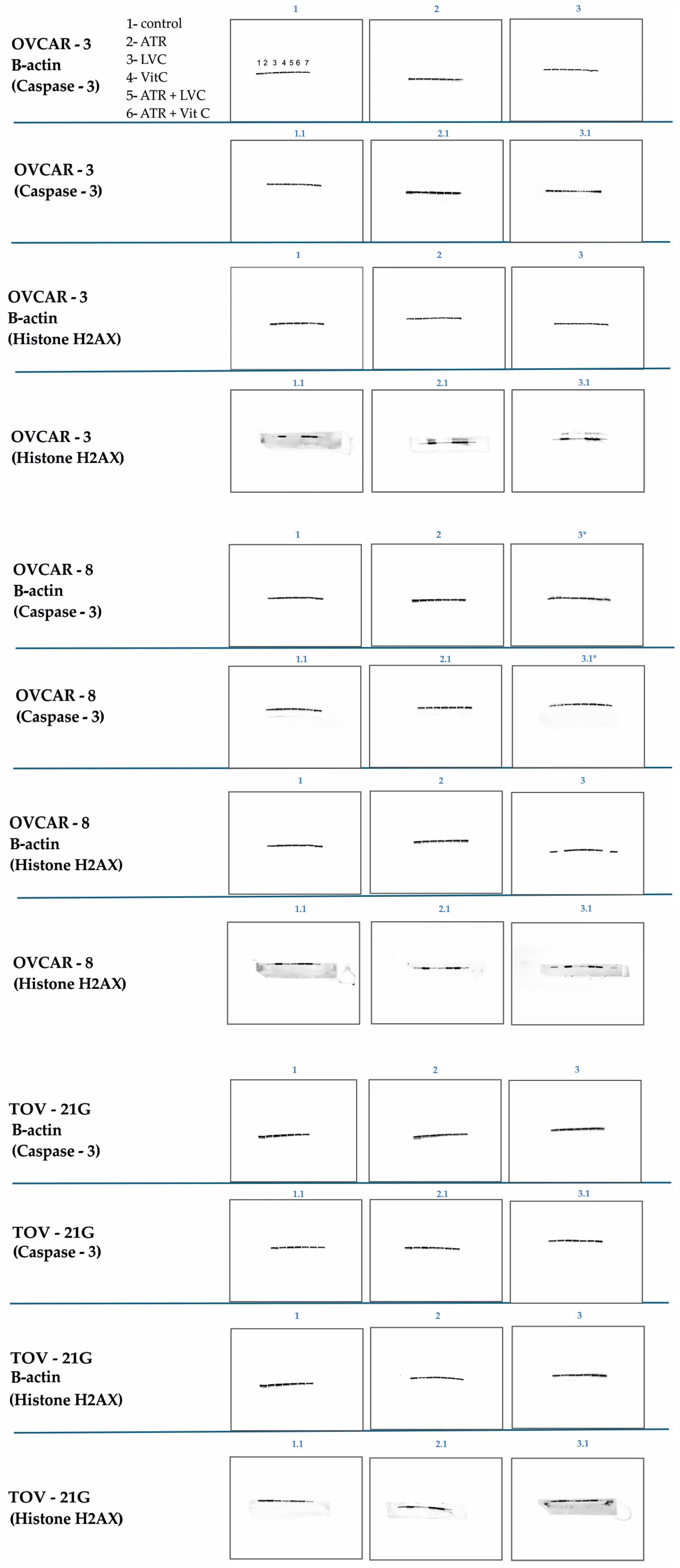
Original photos of membranes. For proteins analysed on individual sections of the same membrane, the same β-actin loading control is presented for all corresponding sections derived from that membrane. * The control sample was loaded in the first two separate lanes.
