# Supplementary material for: Liposomal Vitamin C as a Modulator of the Efficacy of Ceralasertib Therapy in Ovarian Cancer
Source: Int J Mol Sci. 2026 Mar 13;27(6):2630. doi: 10.3390/ijms27062630 (PMC13026461; doi:10.3390/ijms27062630)

Supplementary Information

Liposomal Vitamin C as a Modulator of the Efficacy of Ceralasertib Therapy in Ovarian Cancer

Patrycja Gralewska-Zając [1,†], Aleksandra Przybylska [1,†], Marek Langner [2,3], Magdalena Przybyło [2,3], Agnieszka Marczak [1] and Aneta Rogalska [1,*]

[1] Department of Medical Biophysics, Institute of Biophysics, Faculty of Biology and Environmental Protection, University of Lodz, 141/143 Pomorska Street, 90-236 Lodz, Poland; patrycja.gralewska.zajac@biol.uni.lodz.pl (P.G.-Z.); aleksandra.przybylska@edu.uni.lodz.pl (A.P.); agnieszka.marczak@biol.uni.lodz.pl (A.M.)

[2] Department of Biomedical Engineering, Wroclaw University of Science and Technology, 13 Grunwaldzki Square, 50-377 Wrocław, Poland; marek.langner@lipid-systems.pl (M.L.); magdalena.przybylo@lipid-systems.pl (M.P.)

[3] Lipid Systems Ltd., 48C Krzemieniecka Street, 54-613 Wrocław, Poland

* Correspondence: aneta.rogalska@biol.uni.lodz.pl

† These authors contributed equally to this work.

Supplementary Figure S1 (Figure S1)

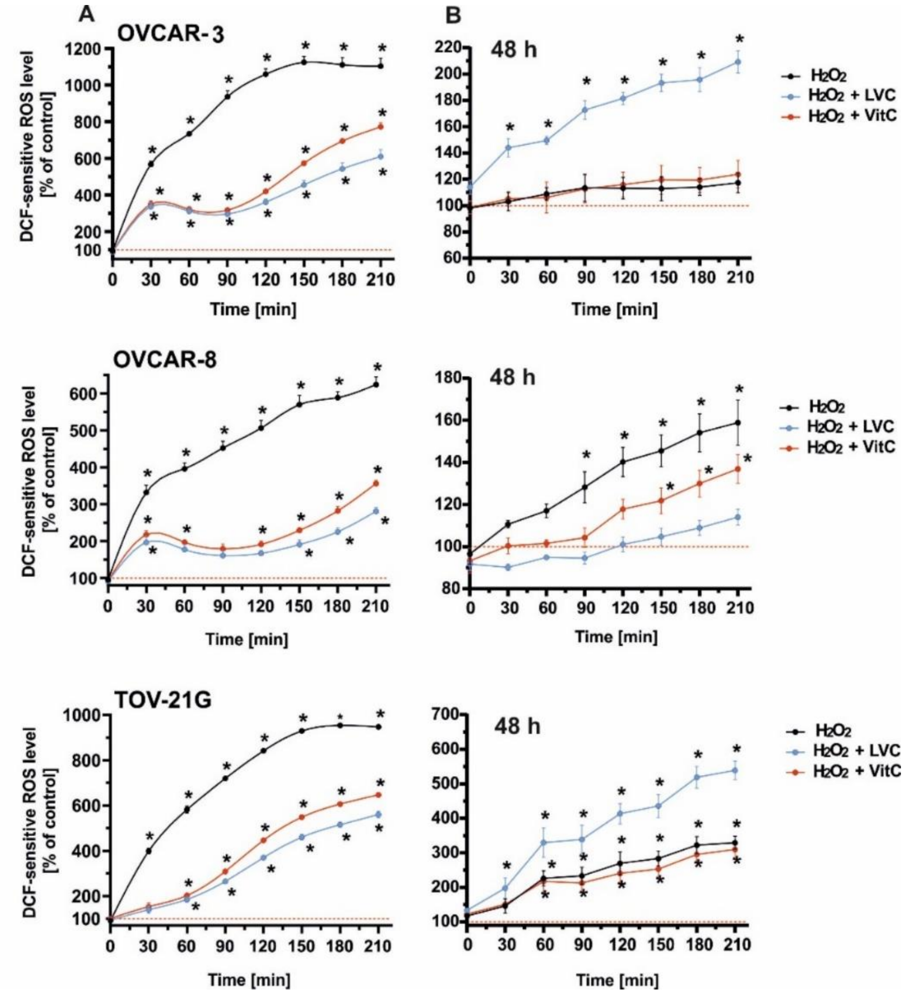

Figure S1 Analysis of intracellular ROS in ovarian cancer cells after treatment with 100 μM H_2O_2 or in combination with forms of vitamin C **(A)** Time-dependent changes in DCF-sensitive ROS production in OVCAR-3, OVCAR-8, and TOV-21G cell lines measured over 210 min. **(B)** ROS levels measured after 48 h of treatment in time. * Statistically significant differences between cells incubated with the compound compared with the control cells ($p < 0.05$).

Supplementary Figure S2 (Figure S2)

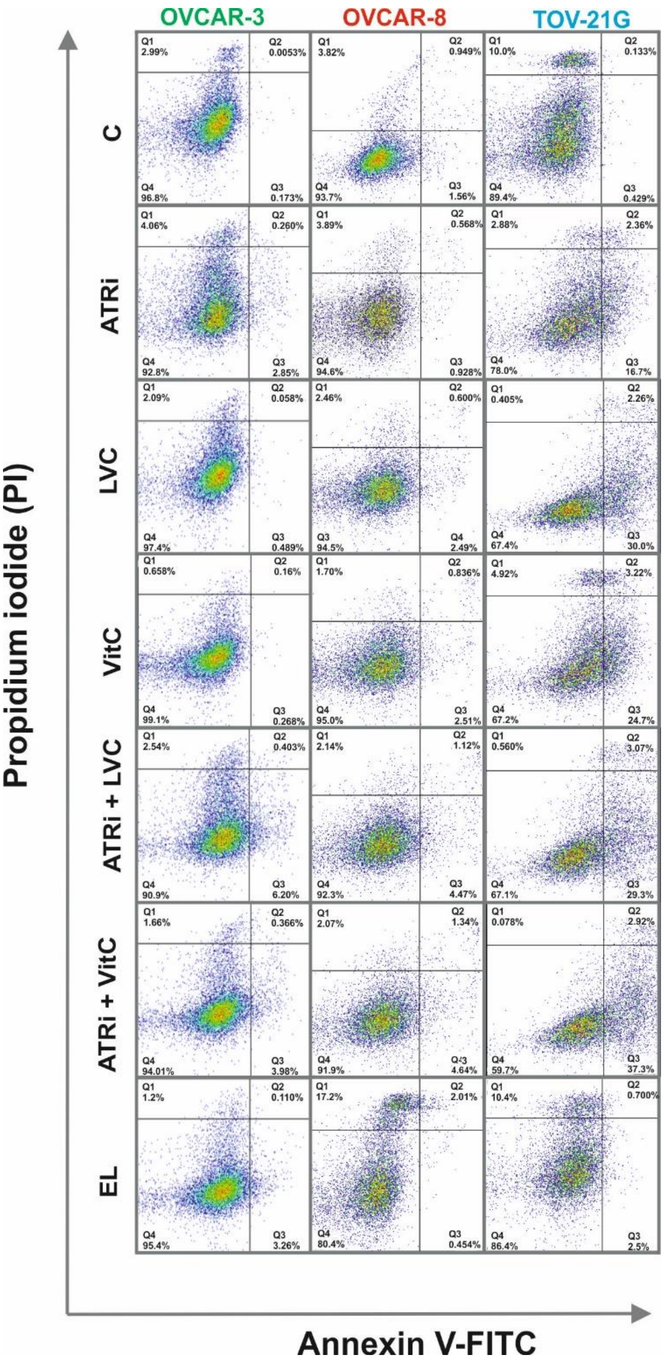

Figure S2 Analysis of apoptosis markers in ovarian cancer cell lines subjected to ATRi, LVC and VitC treatments. Apoptosis was examined via dual staining with annexin V-FITC and PI, quantified with flow cytometry. Representative dot plots of annexin V-FITC and PI-stained OC cells with the indicated percentage of necrotic cells (Q1), late apoptotic cells (Q2), early apoptotic cells (Q3), and viable cells (Q4).

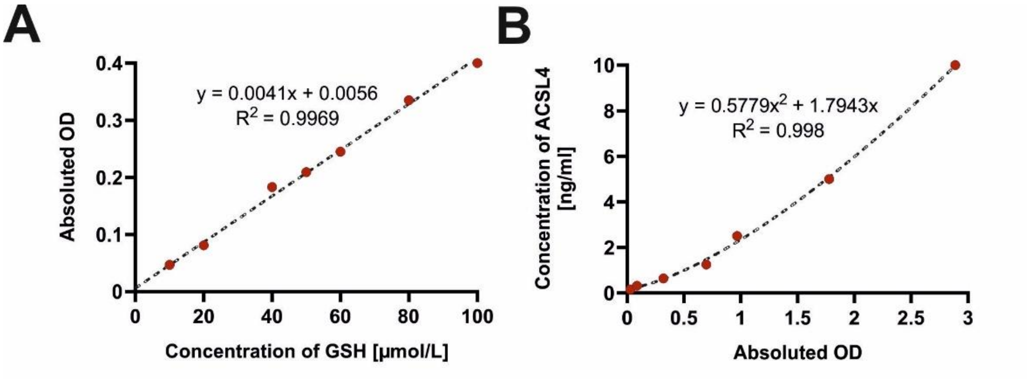

Figure S3 The calibration curve for reduced (A) GSH in the range 0 – 100 µmol/L (B) ACSL4 in the range 0 – 10 ng/ml.

Supplementary Table (S1)

Key materials and reagents used in the study

Reagent	Catalogue Number	Manufacturer
BSA (bovine serum albumin)	A3059	Sigma-Aldrich (Merck)
DCFH2-DA	D6883	Sigma-Aldrich (Merck)
Ceralasertib	TBW02661	Wuhan ChemNorm Biotech
DMEM, high glucose, GlutaMAX™ Supplement, HEPES	32430100	Gibco (Thermo Fisher Scientific)
DMSO (dimethyl sulfoxide)	363550117	Avantor Performance Materials Poland
FBS, heat-inactivated, qualified	A5256801	Gibco (Thermo Fisher Scientific)
H2O2	21;676-3	Sigma-Aldrich (Merck)
HaltTM Protease & Phosphatase Single-Use Inhibitor Cocktail (100x)	78442	Thermo Fisher Scientific
Methanol >99%	BA1990110	Avantor Performance Materials Poland
MOPS SDS Running Buffer	MPM0PS	Millipore (Merck)
mPAGE® 4X LDS Sample Buffer	MPSB	Millipore (Merck)
mPAGE® Bis-Tris Precast Gels 10%	MP10W10	Millipore (Merck)
MTT	20395.03	SERVA Electrophoresis
NAC (N-acetylcysteine)	A7250-10G	Sigma-Aldrich (Merck)
PBS (phosphate buffered saline)	X0515	Biowest LLC
PerceTM Detergent Compatible Bradford Assay Reagent	1863028	Thermo Fisher Scientific
PMSF (phenylmethylsulfonyl fluoride)	36078	Thermo Fisher Scientific
PVDF Membrane	IPVH85R	Millipore (Merck)
RIPA Lysis and Extraction Buffer	89900	Thermo Fisher Scientific
RPMI 1640 Medium, GlutaMAX™ Supplement, HEPES	72400054	Gibco (Thermo Fisher Scientific)
SpectraTM Multicolor High Range Protein Ladder	26625	Thermo Fisher Scientific
Trypsin-EDTA	25200072	Gibco (Thermo Fisher Scientific)

Supplementary Table (S2)

Assay Kits used in the study

**Assay Kit**		
Fluo-4 NW Calcium Assay Kit	F36206	Molecular Probes
Dead Cell Apoptosis Kits with Annexin V for Flow Cytometry	V13245	Invitrogen (Thermo Fisher Scientific)
C11-BODIPY 581/591	SML3717	Sigma-Aldrich, Merck
Cell Ferrous Iron (Fe2+) Fluorometric Assay Kit	E-BC-F101	Elabscience Biotechnology Inc.
Reduced glutathione (GSH) colorimetric assay Kit	E-BC-K030-M	Elabscience Biotechnology Inc.
Human ACSL4(Acyl Coenzyme A Synthetase Long Chain Family, Member 4) ELISA Kit	ELK8993	ELK Biotechnology Co., Ltd.

Supplementary Table (S3)

Primary antibodies used in the study

**Target**	**Host and Clonality**	**Manufacturer**	**Catalogue Number**	**Dilution**	**Dilution buffer**	**Blocking agent**
caspase-3	Rabbit monoclonal	Cell Signaling Technology	D3RGY	1:1000	5% BSA in TBST	Blocking Buffer
γH2AX (Ser139)	Rabbit monoclonal	Cell Signaling Technology	9718S	1:1000	5% non-fat milk in TBST	5% non-fat milk in TBST, Blocking Buffer
β-actin	Mouse monoclonal	Sigma-Aldrich (Merck)	A1978	1:10000	5% non-fat milk in TBST	5% non-fat milk in TBST

Secondary antibodies used in the study

**Species Reactivity**	**Conjugate**	**Host**	**Manufacturer**	**Catalogue Number**	**Dilution**	**Dilution buffer**
Mouse	HRP	Goat	Invitrogen (Thermo Fisher Scientific)	A28177	1:10000	5% non-fat milk in TBST
Rabbit	HRP	Goat	Cell Signaling Technology	7074	1:3000	5% non-fat milk in TBST

Supplementary Figure S4 (Figure S4)

Figure S4. Original photos of membranes.

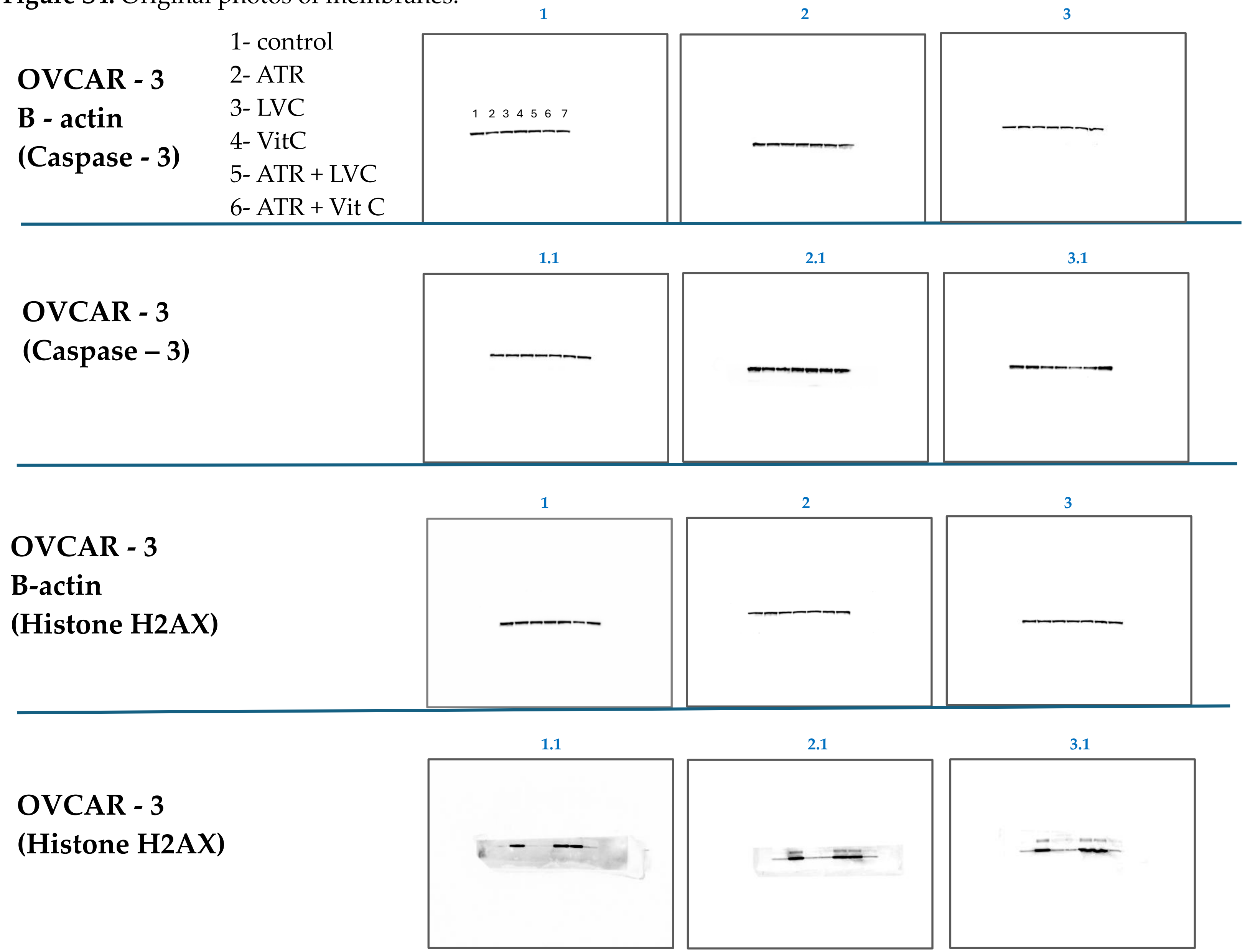

OVCAR - 8
B-actin
(Caspase-3)

1

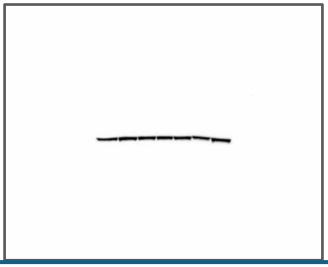

2

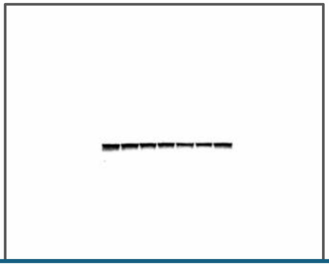

3

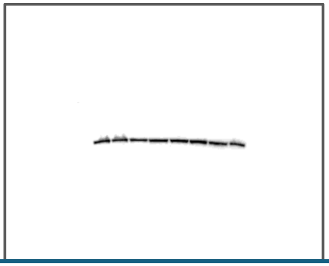

OVCAR - 8
(Caspase-3)

1.1

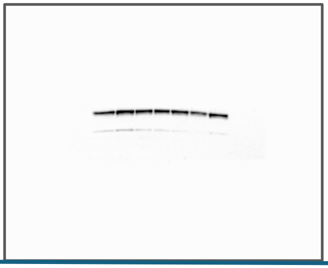

2.1

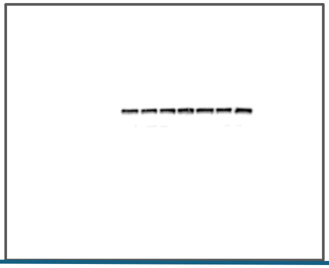

3.1

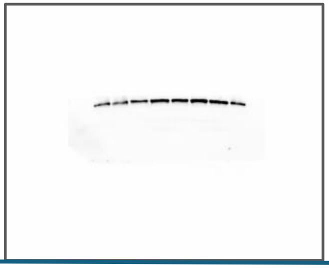

OVCAR - 8
B-actin
(Histone H2AX)

1

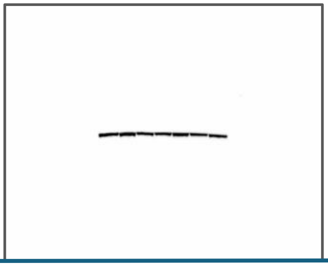

2

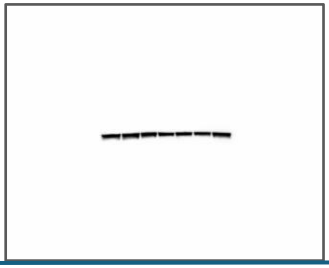

3

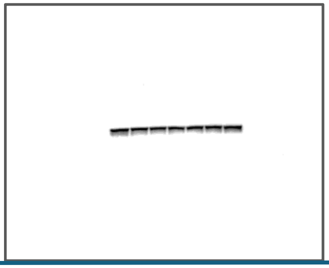

OVCAR - 8
(Histone H2AX)

1.1

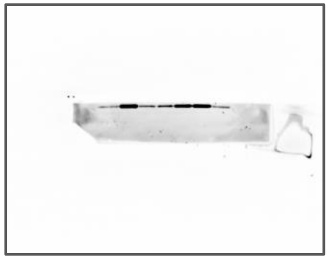

2.1

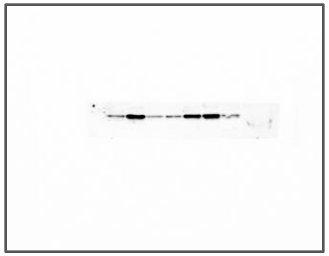

3.1

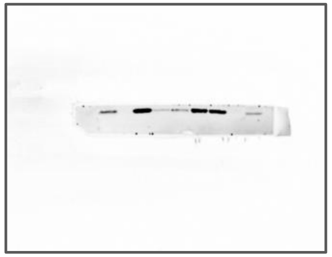

TOV - 21G
B-actin
(Caspase-3)

1

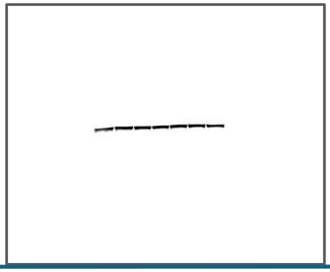

2

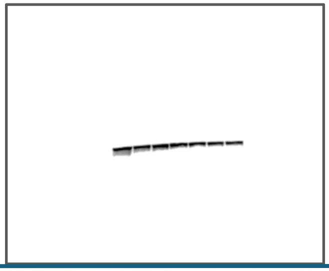

3

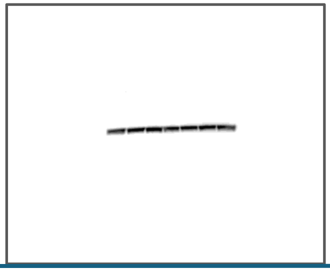

TOV – 21G
(Caspase-3)

1.1

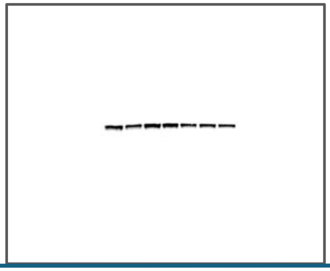

2.1

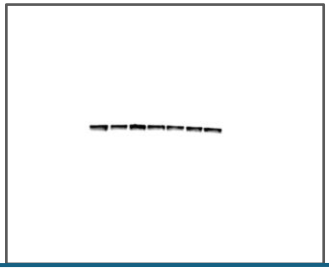

3.1

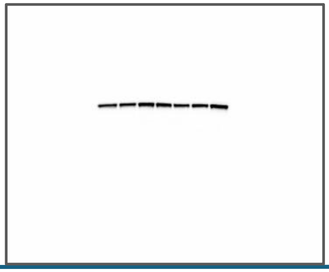

TOV – 21G
B-actin
(Histone H2AX)

1

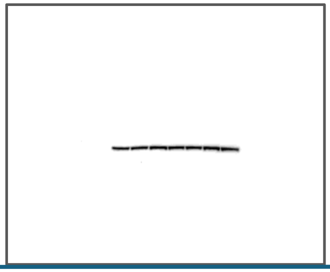

2

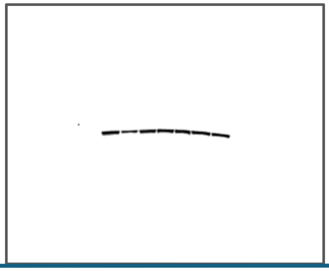

3

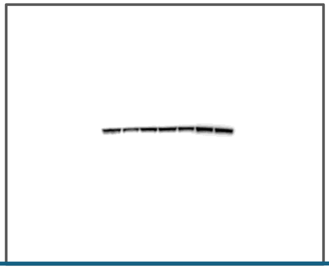

TOV – 21G
(Histone H2AX)

1.1

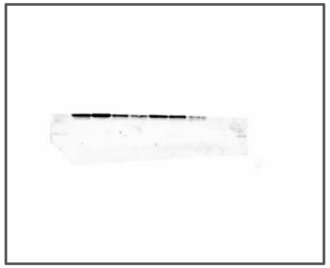

2.1

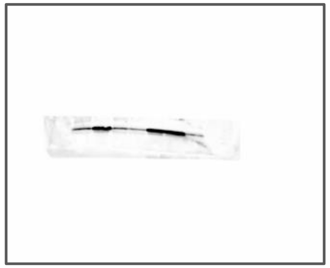

3.1

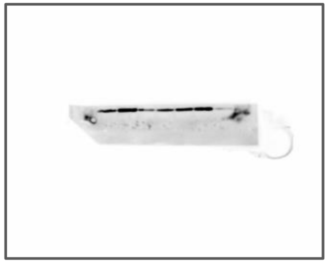

Supplement: Supplementary file 1 [file ijms-27-02630-s001.zip › ijms-4170215-supplementary.pdf]
